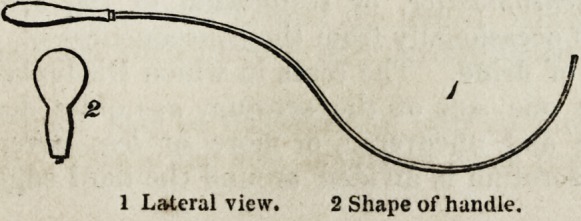# The Operative Surgery of J. F. Dieffenbach

**Published:** 1846-04

**Authors:** 


### BRITISH AND FOREIGN

### MEDICAL REVIEW,

FOR APRIL, 1846.

### PART FIRST.

### Analytical and Critical Reviews.

### Art. I.

*Die Operative Chirurgie* von Johann Friedrich Dieffenbach. Erster Band.—*Leipzig*, 1845.

*The Operative Surgery of* J. F. Dieffenbach. First Volume. *Leipsic*, 1845. 8vo, pp. 856.

There is a great difference between authority in matters of opinion, and authority in matters of fact. Combating as we have continually done against the evil effects of the former in impeding the progress of inquiry, we have been equally willing to yield a ready deference to the latter. Medical authority and surgical authority, also, must be regarded in a different light, even in practical matters. The effects of medicines are obscured by the natural progress of the disease; by changes of weather, and by moral and other influences often inappreciable to the physician, so that some scepticism becomes natural to the critical inquirer after truth, when comparing the results of opposite modes of treatment in internal diseases. But in surgery the nature of the cure is plain, the end attained by an operation evident, and when a man whose practice is open to the scrutiny of a public hospital comes forward and says, "I have performed this operation a thousand times, and prefer this plan," every prudent surgeon must bow to such authority, and feel grateful to the writer who thus enables him to correct or modify the impressions derived from his own more limited experience. Regarded in this light the work before us is one rather for analysis than criticism. The author states that he has written his book "to communicate what he has found useful in surgery to those who have seen less than himself,"—like Richter, desiring not to write a book for the learned, but one containing no page in which a practical surgeon may not learn something calculated to prove useful at the bedside. He says he has seen almost everything that can happen to the sick, and has observed with his own eyes, and never through "coloured or strange glasses," and in all cases has preferred expressing his opinion freely and openly, to taking a "half-and-half course between yes and no." The work is no survey or retrospect of an anxious and busy life, no pensive contemplation in the evening of existence, but the record of daily observations made with all the ardour of youth in the presence of passing events. It is replete with valuable practical matter, the more important part of which we now proceed to lay before our readers.

After a few introductory pages, and a very hasty glance at the history of operative surgery, with some general remarks on surgical instruments, to the end that they should be of the best material, and simple in construction, the operator working and not the tool—our author enters upon the first division of his work, a description of the "operations which are performed on the various parts of the body." The whole of the volume before us is occupied by this division, extending to sixty-seven chapters, which we now proceed to examine in detail. The First is on the extraction of foreign bodies, and is divided into four sections: 1. On the removal of foreign bodies fixed to, or surrounding projecting parts of the body, as rings over the fingers or penis. 2. On their extraction from the natural passages, nares, antrum, meatus auditorius, eyelids, air-passages, pharynx, and œsophagus, stomach and intestines and rectum, and genital organs of the female. 3. On their extraction from the substances of the body, as balls and shot, parts of knives, nails, glass, &c. 4. Method of removing foreign bodies which remain after the healing of the wound caused by this introduction: here we have a curious case of a girl who had been subject to epileptic fits, which ceased after the removal of several fragments of glass from the hand by incision, which had remained several years, she having broken a glass in her hand. The chapter is closed with remarks on the removal of fragments of dead bone, and is useful and practical, without however containing anything particularly new, or peculiar to the author.

The second chapter is on *the surgical knot,* or the union of wounds by suture, comprehending the treatment of wounds by the common suture with the assistance of adhesive plaster, &c., the various applications of the twisted suture, and a comparison of the advantages of the two methods. The latter is a great favorite with Dieffenbach in plastic operations, and in all cases where very accurate union is required to obviate ugly cicatrices. He employs the small spring-hardened insect needles, manufactured at Carlsbad. The use of the subcutaneous suture is then recommended in cases of obstinate salivary, urinary, and other fistulæ. The thread is introduced beneath the skin at some distance from the fistulous opening, and the canal is surrounded by the ligature in the same manner that an artery is tied. The ligature may be left from four to fourteen days, according to circumstances; it is better to withdraw it too late than too early. It is to be employed if the twisted suture have failed, and the author states that he has often used it with success in cases which had resisted every other mode of treatment. The chapter is concluded by some remarks on the knots of the olden surgeons. The author does not appear to be acquainted with the platinum suture, so successfully employed at Guy's Hospital by Mr. Morgan.

In the third chapter *the potential and actual cautery* are described, their uses and modes of application. The chapter is long, but the author expresses himself as decidedly opposed to burning in any way when other means are applicable, and states that this practice has much declined in Germany.

*The application of leeches* is the subject of the fourth chapter, venesection of the fifth, and arteriotomy of the sixth. They are all good, but do not require remark from us, nor do the two following on infusion and transfusion. In the former the methods of injecting various saline and other solutions into the veins are described, and in the latter the blood of one person into the veins of another.

The ninth chapter treats on *the ligature of arteries.* The general remarks on the application and effects of the ligature to suppress bleeding from wounds and obliterate the trunk in states of disease, are concise and practical, but somewhat commonplace. The operations upon particular arteries are then described, namely, the common carotid, superior thyroid, lingual, temporal, occipital, posterior, auricular, innominate, subclavian and axillary in various situations, inferior thyroid, internal mammary, brachial, ulnar, radial, abdominal aorta, with its subdivisions, and the arteries of the lower extremities. In a work from the head of the German school of operative surgery, professedly a complete system of this department of medical science, one would naturally expect to find such an important subject as thel igature of arteries fully and completely treated, or, to say the least, that some results of the personal experience of the writer would be given for the guidance of his readers in their operations upon the principal arterial trunks. He states that what the discovery of printing was to science, of gunpowder to war, of railroads for the communication of one people with another, the discovery of the ligature of arteries was to surgery; yet he describes the methods of tying all the arteries in the body separately in eighteen pages. The innominata occupies half a page, the internal and external iliacs the same space, the aorta a little more. We should not quarrel with this if he had condensed what was known on the subject into this space, or if he had given a clear account of what he had found to be the best mode of operating; but instead of this we have a hasty and imperfect sketch of the anatomy of the parts concerned, and of the different methods adopted by several surgeons, without appreciation of their respective merits. The chapter is altogether unsatisfactory, and we suppose does not come within the writer's *spécialité.*

In the following chapter on *the torsion of arteries,* Dieffenbach informs us, that, like other surgeons, he has found it useful in checking the hemorrhage from small vessels bleeding upon an open surface, as after the removal of tumours. With regard to torsion in the continuity of an artery, instead of the ligature to produce its obliteration, he well remarks that, however interesting as a physiological experiment, it is quite useless to the practical surgeon.

The eleventh chapter treats of the *operation for aneurism* after the methods of Antyllus, Hunter, and Brasdor, the history of these methods and the cases in which they are severally applicable. The author considers that of Antyllus the best in cases of false aneurism following wounds of arteries, as in the elbow after unskilful venesection. We may remind our readers that Antyllus's plan is that of tying the vessel both above and below the aneurism, and then opening the sac. In all cases of true aneurism this is to be discarded for either Hunter's or Brasdor's operation. The operation for false aneurism is first treated in general, and then when situated in the bend of the left elbow. This is minutely and well described, but appears to have been far from successful, as the author states that he has very frequently performed it, after wounds from venesection, and notwithstanding the most careful operating and after-treatment, at least a fourth have died. He goes on to the consideration of popliteal aneurism and its treatment by ligature of the femoral artery, and then to the operation of Brasdor. This he considers of great value in cases where it is impossible to place the ligature between the aneurismal sac and the heart. The ligature being placed beyond the sac, the blood cannot flow from the latter; it stagnates there, coagulates, afterwards becomes absorbed, and the sac is necessarily obliterated. The ligature must not be placed too near the sac, or the diseased coats of the artery will give way, and secondary hemorrhage be the result. This is of course all well known, but it is well to know the present opinion in Germany on so important a subject.

*The operations for varicose tumours.* These are considered in the twelfth chapter. Exposure of the tumour by incision without opening it, and filling the wound with charpie, which heals by cicatrization, is not dangerous, but very seldom successful. The same may be said of puncture with the lancet, and compression afterwards by plaster and bandage. Incision is more dangerous without being more successful. Extirpation has been recommended by exposure of the varix and division of the veins leading to it, above and below. It is also dangerous. Ligature of the vein in any way is highly dangerous. Ligature by needles and twisted suture, as recommended by Velpeau, Dieffenbach says he has several times seen followed by great danger in Paris. The carrying a needle and thread through the tumour, and leaving the latter for two days, has been followed by obliteration of the veins, but also by severe phlegmonoid inflammation. Cauterization of the varix is described as excessively dangerous. On the whole, the author is of opinion that no patient should be recommended to put his life in danger by undergoing any of these operations, or any others which have been proposed, in order to get rid of what is seldom more than an inconvenience. The disease is generally a constitutional one, and operation is no more to be recommended for a single varix, than for an external scirrhous tumour, when the same disease exists in other parts of the system. We believe that this is the conclusion to which the experience of the last twenty years must bring all prudent surgeons. We have seen the application of caustic potash along the course of the vein above the varix extensively used. The vein participates in the inflammation of the surrounding parts, and generally just enough so to cause its obliteration without being followed by diffuse phlebitis. A succession of forty successful cases, without a single symptom of dangerous consequences, had led us to believe that at length the true method of treating varix was discovered, when two fatal cases in succession dispelled the delusion, and we believe, as we have just stated, that it is better to diminish the inconvenience of varices by laced stockings or elastic bandages, than to recommend any operative proceeding whatever.

The author scarcely touches upon the mechanical treatment of varix; yet much more than palliation of distressing symptoms may be obtained by its judicious application; and we believe in stating the result of our observation of the result of simple compression in the treatment of varicocele, we impart a practical fact of great value to our readers. The unsatisfactory results of all the known methods of treating this disease, with the exception of the ligature of the veins, which though often successful, is often followed by dangerous phlebitis, led us to reflect on the effects of the ligature and the means of safely fulfilling the same indications. What is the effect of the ligature when successfully applied? Simply obliteration of the vein, preventing the gravitation and pressure of the column of blood upon its dilated portions, and necessitating the return of the blood by some other channel. Now came the question, how could compression be applied to fulfil the same purpose? At first sight it appeared that anything like compression between the varix and heart would inevitably increase the tumour by preventing the veins from returning their blood to the heart; but still a ligature does so, and if it does not kill the patient, often cures the disease. We determined then to try the effects of compression upon the spermatic veins as they pass through the inguinal canal, and at first used linen compresses and bandages, with some encouraging result, but a little experience proved that a common truss was by far the most convenient means of applying pressure. We have notes of 24 cases of greater or less severity in which wearing a truss from 6 to 18 months has completely cured varicocele of long standing. The patient should be kept in bed or on the sofa, with the scrotum elevated during the first week of its application, and the pad applied immediately above the pubes, so as to press upon the whole of the inguinal canal. The pad must not be too large and flat, or it will not answer the desired purpose; nor too conical, or it will not only cause inconvenience and pain, but also, by separating the fibrinous insertions of the oblique muscle, reduce their resistance to herniary protrusion. The common spring-truss, well fitted, answers much better than the ball-and-socket pad, and has not the disadvantage of the pad behind. Immediate relief follows the application; the column of blood is prevented from bearing upon the dilated veins; they contract, and the portions subjected to pressure become either greatly thickened in their coats, as their hardness evinces to the finger, or perhaps altogether obliterated. We have not hitherto had an opportunity of examining the state of the veins after death; therefore do not enlarge upon the anatomical effects of this mode of treatment, but strongly recommended it as a simple, safe, and successful substitute for former difficult, hazardous, and doubtful proceedings.

In the following nine chapters, vaccination, cupping, scarification, the formation of issues and setons, the opening of acute and chronic abscesses, simple and electro-galvanic acupuncture, and the dilatation of wounds, are successively treated. We have found nothing on any of these subjects peculiar to the author or to German surgery, and therefore pass to the 22d chapter.

*The operations upon cicatrices.* Operation upon a cicatrix may be required,—1, when it has disfigured the form of any part; 2, when it interferes with the function of a part; 3, when its condition is morbid.

The operations are four—1, subcutaneous division; 2, division from without; 3, extirpation; and 4, incision or extirpation, with transplantation of skin.

Subcutaneous division is described as often successful in removing contractions of cicatrices about the face, neck, arms, and fingers. It is performed with a fine, narrow, sickle-shaped knife. At any convenient point of the cicatrix, the neighbouring healthy skin is punctured; the knife is passed beneath the base of the cicatrix, which is then divided as freely as possible without injuring the skin. In cases where the skin and subcutaneous tissue have become adherent to subjacent periosteum, the knife is passed flatly as near the bone as possible, and division effected to any extent which may be necessary. If the cicatrix be very large, it may be necessary to make more than one puncture in the skin. The cicatrix only should be divided, not any of the surrounding healthy cellular tissue. If bleeding beneath the skin come on, charpie and compresses must be applied, and the part surrounded by a bandage. This general description of the operation will apply to all cases in which it may be necessary to remedy distortions of the mouth, nose, eyelids, neck, fingers, &c.

The division of a cicatrix from without is seldom necessary to remove disfiguration of a part, but often to restore the functions of a limb. The operation consists either in a simple transverse division, in repeated transverse separation or notching, or in oblique division. The first plan is often successful in sharp band-like cicatrices in the neck and flexures of the limbs; the second, when the cicatrix consists of indurated cellular tissue, firmly adherent to surrounding parts, and particularly when it is connected with a tendon; the third is most useful after wounds in the inside of the hand and fingers, and is generally successful. Dieffenbach has completely restored the use of the finger after it had been so drawn into the palm of the hand as to fix the nail in the skin, and cause the patient to pray for amputation.

The *extirpation* of cicatrices is particularly required in young females to diminish disfiguration, and in other cases where its texture is morbid; when the wound was produced by broken glass, china, &c.; in cases of hypertrophy of the cicatrix, or chronic inflammation, with re-opening, which resists other treatment. Incisions are made in the healthy skin, including the whole extent of the cicatrix, which is then raised with forceps and dissected away, and the edges of the wound brought into close apposition by small needles and the twisted suture. If, however, the cicatrix be so large that the union of the edges of the wound would be impossible, repeated partial extirpation is the best practice. A long slip, with pointed edges, should first be excised from the middle, and when the wound so produced has healed, after some weeks, or, better still, some months, another piece is excised, and so on until the whole has been removed.

In some of the most difficult cases it is necessary to divide the cicatrix in its centre, and transplant skin from a neighbouring part between the edges of the wound. The manner of doing this will be explained in the division of Plastic Surgery, and we may refer to Dr. Müller's case, given in our 19th vol., p. 411-12. In all cases, after these operations, warm applications are far preferable to cold, as chronic inflammation, lividity and death of the edges of the wound frequently follow the latter.

*Operations on nævi.* In this chapter the author speaks highly of the effects of the pure liquor plumbi, and of the solution of alum, in flat nævi up to the size of a crown-piece. Lint steeped in the lead solution is fastened over the part with a bandage, and the lint wetted by fresh applications of the lead, without frequent removal of the lint. After days, or weeks, the swelling becomes whiter, flatter, and firmer; soon after, little firm white spots form on the surface, and the cure is certain. By means of the solution of alum and compression, Dieffenbach has cured nævi so large that extirpation would have been impossible. It may be necessary to keep it constantly applied for six months.

Compression is often useful, but often also dangerous, and frequently inapplicable from the situation of the nævus.

Irritation by blisters, vaccination, &c., has never proved successful in the experience of the author.

Tattooing with three needles, bound together, and dipped in a coloured solution, which is also rubbed in, has been said to alter the colour of the spots. The author does not speak of its effects from personal experience, but says it may be employed on the faces of young females, where other means are inapplicable. He does not give the composition of any of the coloured fluids.

Transfixing the tumour in various directions with needles is useless in the opinion of our author: it is very uncertain even when threads are carried by the needles, and then tied. The seton is not to be recommended.

The ligature is to be applied when the tumour is pediculated, or when situated in the mouth, neck of the uterus, &c. If the base be broad, needles may be passed through it, and the threads knotted in opposite directions.

Caustics aud cauteries are generally more painful, and less useful than excision.

Ligature of the principal artery leading to the tumour is not to be recommended, as its result is very uncertain.

Extirpation of the tumour and union of the edges of the wound by needles and twisted suture, is the best method of all, when the lead or alum has failed. Extirpation is to be either total or partial, according to the size of the tumour. If partial, it is done in the same way as partial excision of a cicatrix. Several interesting cases are given as examples of the success of this method: the chapter is a practical and interesting one.

*Operations on tumours.* We now come to the operations for the removal of tumours. These are,—1, extirpation by the knife; 2, ligature; and 3, chemical and mechanical means for their destruction. Of the two former methods we have nothing new in the work before us, but several cases are recorded, in which large encysted tumours, containing fluid, were successfully treated by trocar puncture, and carrying a seton through the tumour. This practice is particularly recommended in large watery tumours with thin sacs, lying between muscles, and especially between the muscles of the back, and beneath the scapula. The trocar is introduced, and the seton passed through the canula, which is then withdrawn. The seton is smeared with the cantharides ointment, and several times daily it is partially withdrawn, the wet portion cut off, and all discharge squeezed from the sac of the tumour. When the discharge becomes thicker the seton is withdrawn, the openings only kept open, and compression applied to the other part. Caustics are occasionally useful to destroy parts of cysts which cannot be removed by the knife.

In chapter 25 we find the operations for *ganglia* described. Dieffenbach finds the best treatment is to burst them by a smart blow with a hammer. This would frighten most patients a great deal more than the plan usually followed in this country, of striking with the back of a book. We should think it also very easy to miss the aim with a hammer, and probably strike the wrist instead of the ganglion.

*Tumours of nerves* are to be removed by incision, and exposure of the nerve above and below the tumour as far as its size is increased. Where it is seen of the natural caliber, it is to be divided, and the enlarged part, with the tumour, dissected away together.

We have now a very long chapter on *polypi.* Various operations are required, according to their nature. Caustics are principally applicable to carcinomatous polypi; extraction to polypi of mucous membranes; ligature to fleshy vascular polypi; and excision to those of a fibrous texture.

The rules for the treatment of nasal polypi do not differ from those laid down in most surgical works. Several interesting cases are recorded in which large fibrous polypi of the fauces were removed by excision, division of the velum palati being necessary before they could be reached or removed. The fissure in the velum was afterwards healed by suture.

The various modes of operating upon polypi in all situations are fully described in a sound practical manner. The chapter is a useful one, but does not add much to our former knowledge of the subject.

PLASTIC SURGERY. We now arrive at the principal subject of this volume, and favorite study of its author—Plastic Surgery, which is treated at great length, occupying upwards of seven hundred pages. It is an amplification of his former work on the Restoration of Lost Parts, which we noticed in our Seventh Volume (pp. 386-416), in conjunction with the treatises of Blandin, Zeis, and Liston. The barrenness of our own literature in this department of surgery we endeavoured in some measure to supply by the article just referred to, and in a more recent one on the works of Von Ammon, Baumgarten, Serre, and Mütter (vol. XIX, p. 396-427); but it induces us again to enter at some length into the description of curative measures, which certainly have not received their due share of attention from surgeons at large. We do so the more willingly, as eleven years have now elapsed since the former work of Dieffenbach was published, and during this period he has enjoyed extensive opportunities of testing the merits of his former proceedings, of modifying them, or substituting others which may be preferable. Our remarks must be regarded rather as an analysis or condensation of the statements of the author, than a critical examination of his respective operations, which must necessarily be more fully tested here, before such criticism would be of much practical value. We proceed, therefore, simply to give an account of his present views and operations, merely omitting those points which have been already discussed in our former articles, to which we would recommend the student again to refer.

Plastic surgery is defined as consisting in "the replacement of a lost, or the restoration of the form of a mutilated (verstümmelten) part of the human body." If "imperfect" had been added to mutilated, this definition would have been more correct, as many of the applications of plastic surgery are required for the relief of congenital deformities, which can scarcely be said to be mutilations.

The history of plastic operations is fully entered into, but our first article renders it unnecessary here to follow our author. He considers that the French remain far behind the Germans, and describes Blandin's *Autoplastie* as "an empty compilation, in which the author appropriates foreign property to himself." (p. 317.) Serre's work is not much better, and many important things are unknown to him, while Labat's contains much interesting matter. It is well to know what one great surgeon thinks of another; but we cannot admire the uncourteousness which we remark, not only here, but frequently in the writings of the German when speaking of his contemporaries. Very little ceremony is used with opponents or rivals, while flattery of a somewhat coarse description throws a questionable halo around friends and supporters.

The basis of all plastic operations is, that separated portions of integument unite to fresh surfaces of wounds of other parts, when, by means of a small band, the nervous and vascular communications are kept up, or when the separated portion retains for itself a high degree of vitality. Having before described the consecutive conditions of the flap after its elevation and adjustment, we extract some important remarks upon the various forms and changes in its texture, with respect to various modes of operating:

"The principal efforts of the new formed part are directed towards its limitation or isolation. The transplanted superficial flap contracts concentrically, drawing the borders together, and resembles a rounded hillock. The degree of elevation of the centre and furrowing round the borders depends upon the rigidity or yielding nature of the surrounding parts. The elevation is even level in cases of transplantation into fixed skin; when into the yielding skin of the eyelids, it almost resembles a ball; and remains perfectly flat when into a thin skin, which extends over a flat bone. When the flap is fixed in a raised position, so as to resemble a sort of roof over an opening, its under surface does not become covered by skin, but the two parts either adhere together, or approach each other, and granulations fill up the intervening space; the nose also becomes massive, and its openings only remain open, when the skin falls inwards.

"When the flap is applied upon a superficial fresh wound, it unites both with the borders and the base.

"When the flap is applied to a part covered by skin, as to the stump of a nose, so that the wounded cellular tissue of the flap lies upon the sound epidermis, after the union of the edges the following changes occur: The cellular surface is not covered by skin, but becomes smooth, pale, and receives a fine polished transparent exhaling membrane; the opposed epidermis of the stump receives a similar moist separate covering, and the two surfaces remain for a long time like the two opposed surfaces of a serous membrane—the pleura of the lungs and thorax, for example—separated by the exhalation. When the access of air is prevented, no suppuration occurs, but the cellular surface and that of the epidermis unite together as soon as they perfectly resemble each other. The improvements I have lately undertaken upon noses formed by art, have given me numerous opportunities for pursuing these inquiries.

"If two surfaces of epidermis are brought together, and they are deprived of air, they begin to exhale, and become like serous membranes, uniting, however, afterwards.

"When a serous membrane is fixed outwards, exposed to the air, it is placed in highly irritating circumstances, and changes with difficulty into a dry membrane like epidermis.

"When a mucous membrane is turned from within outwards, it often, in a few weeks, assumes the nature of the external skin, and becomes pale and dry.

"When a flap is perfectly reversed, so that the cellular surface is turned outwards and the epidermis towards the face of the wound, and the edges unite, the following changes happen. At first the flap suppurates, but languidly and thin, (the discharge ?) the surface becomes smaller, the borders contract together, it becomes rolled inwards, like an encysted tumour, both the internal surfaces go on as before described, exhale, and remain a long time separated, until the cellular and epidermic surfaces become united together. A contracting process of cicatrization covers the external wounds and draws the surrounding borders so strongly together, that the cicatrix has scarcely a quarter of the circumference of the wound, in the depth of which the flap lies like a ball. But when a flat bone lies beneath the wound, the cellular surface arches itself strongly outwards, and appears like a lump (buckel) covered by skin.

"The most common property of transplanted parts is, that they appear harder, almost cartilaginous, and afterwards become softer, and often decay, a peculiarity which is brought about by changes in the cicatrix." (pp. 322-4.)

For further remarks on the general principles of these operations, Dieffenbach refers to his former work, and we to our former articles, and follow his account of the rhinoplastic operations. His first chapter is on the formation of the nose from the skin of the forehead, and, as our former articles do not contain a detailed account of this, the grand operation of plastic surgery, we think it well, at the risk of being a little diffuse, to give at full length the directions of one who probably speaks after greater practical experience than any living surgeon can boast of.

THE NOSE. The operation for the formation of an entire nose is a great modification of the Indian operation, and of that of Tagliacozzi, Carpue, &c., because, in all their cases a stump remained, and the operations were undertaken to restore the cartilaginous part of the nose. It is described by Dieffenbach as follows:

"*Preparation.* A nose is to be cut out of a piece of leather, the inner side of which is to be covered by a thin layer of plaster; the form of this leather flap must be almost triangular, and under its middle a quadrangular flap is applied, an inch in length, and three quarters of an inch in breadth. This latter part is the model of the septum. The model, particularly when the skin of the forehead is thin, must be almost a fourth part larger than the nose which is intended to be formed. Several small, delicate-bladed scalpels, hooked forceps, a pair of pincers to nip off the ends of the needles, fine insect needles, curved needles threaded with waxed silk, two measures (Spanne) of long thick cotton threads, slips of plaster, small Indian rubber tubes, soft sponge, &c., must be ready. Several practised assistants surround the operator.

"Before the operation is begun the leather model must be placed on the part, and its borders attached around the stump (by the plaster). If its form is satisfactory, the model is so extended upon the forehead, that the part which is to form the septum is laid close to the hair, which has been previously shaved, in order to prevent its introduction into the wounds.

"At the operation the patient is seated, the back of his head supported against the breast of an assistant, who also supports the side of the head with the palm of his hand.

"1. *The wound of the stump of the nose.* The nose is entirely wanting; a round hole occupies its place, and above this the skin lies flat. The knife is inserted near the right internal angle of the eye, and drawn downwards, and somewhat outwards, towards the commencement of the upper lip, where the ala is placed. A second similar incision is made on the opposite side, which passes obliquely outside the root of the nose in the first descending cut. This is exactly the line from which the nose formerly rose. A third penetrating incision is made across the upper border of the lip, quite down to the bones. The skin between this and the descending incision remains undivided. These three incisions isolate the flat remains of the old nose and the hole in the middle, and remain under the new nose. All the external borders of skin with which the flap of the forehead is to be united must be loosened to the extent of several lines, that they may be readily adapted to the flap.

"2. *Incision of the skin of the forehead.* The knife is inserted near the point of the right side of the model, and, following this, the skin is divided by gently drawing the knife from above downwards, until it arrives at the uppermost point of the right incision. Then the incision on the opposite side is made, but this must terminate a full finger's breadth earlier, so that the skin between the incision on the opposite side of the stump remains undivided, in order to form the bridge. Then the septum is to be cut from the highest part of the forehead, making first the lateral incisions, and lastly the upper transverse one. If this incision were made first the flowing blood would interfere with the formation of the flap. During these incisions the assistant stretches the skin of the forehead by drawing the skin of the temples towards himself. All the corners and angles of the flap must be divided with great care, and before this is effected the loosening of the flap must not be commenced. The upper border of the skin destined to form the septum is fixed by hooked forceps, and separated from the pericranium by drawing the knife freely with its flat surface towards the forehead; the body of the large flap is to be separated in the same manner; and lastly, the bridge is loosened underneath, in order that it may be readily turned.

"3. *Union of the wound of the forehead.* This must take place before the fastening of the flap, partly for the protection of the denuded frontal bone, partly to give time for the troublesome bleeding to cease. The thick edges of the wound between the eyebrows are to be united by several strong points of interrupted suture, proceeding upwards, until stretching of the skin of the forehead commences. Then no more sutures are to be inserted. Then the angles which were produced by the formation of the septum are to be pierced once or twice, and a stitch also placed in each corner between the flaps of the nose and septum. The opening which then remains must be filled with charpie, and strips of plaster brought across the forehead from one temple to the other, to support the sutures.

"4. *Fastening of the flap.* This is effected by means of a number of small insect needles, the ends of which are cut short off. The union must be quite exact in every point. *Some* sutures are also placed between the needles, but *many* are apt to be followed by eversion of the edges and gaping of the intervening space. Lastly, the septum is to be fastened to the upper lip by three very thick sutures, of which the middle one is to be the first applied. The wounded edge of the upper lip is to be curved outwards by means of the index finger placed within the mouth; both edges are to be transfixed, and brought in direct apposition with each other by connecting knots. By this means surfaces of epidermis are prevented from remaining in a corner, and separating the wounded surfaces from each other. The needles are to be snipped short off. The careful pressing together of the partition is now of particular importance, and this is effected by means of a wick formed of twelve threads of thick cotton laid together, which is passed in at one nostril and out at the other, and then generally tied with thick knots. Formerly I stitched the edges of the septum together, and then passed a long strip of plaster around it, but the wick answers better on account of its equable and gentle pressure.

"After the operation is terminated, the nose is to be syringed and cleansed from blood, and light charpie is to be carried under its upper part by means of forceps, in order to prevent any sinking inwards; thick quills with charpie wound round them, or the useful elastic tubes of Zeis, are then to be introduced. The after-treatment will be presently considered.

"*Modifications.* Various complications which are observed when the nose is totally deficient, render a particular kind of incision of its bone necessary. The lip is occasionally drawn by the process of cicatrization into the large hole in the middle of the face, so as to form two oblique limbs (schenkeln) over the bare and dry alveolar process, part of which is often destroyed by necrosis, and sometimes it is so completely turned up as to resemble a total ectropium. With this there may be great loss of the soft parts of the middle of the face, the hole surrounded for a considerable breadth by fine red cicatrices; and to complete the evil, both the under eyelids may be drawn down to the hole by cicatrices and turned outwards.

"In order to replace such a loss of substance, almost the whole integument of the forehead would be required, and the danger to life after the operation would be greatly increased. The nose itself also would always be too small, even with the largest possible flap; and the flap, from the distance of the sound skin of the cheeks would only resemble a flat roof over the hole.

"In such cases I have adopted a proceeding by which the operation is much diminished, while the secondary ectropion of the upper lip and of the eyelid is removed, or the subsequent healing at any rate effected. Loosening of the skin of the face does this.

"After the incisions for the nose-flap have been made in the healthy skin of the face, the upper lip is fixed, and freed from the bone until it can be freely drawn downwards; then the knife is carried upwards under the skin of the face, and this is loosened (demaskirt)." (pp. 327-30.)

In this way, by dividing the subcutaneous tissue, large flaps may be obtained, which may be drawn in almost any direction, and fixed by needle and suture. The mode of their application will be described when speaking of the restoration of lips and eyelids.

When the skin of the centre of the forehead has been destroyed by necrosis of the subjacent bone, the flap must be formed from the side, or one flap from one side and one from the other; or if no healthy skin remain on the forehead, then the skin of the arm must be employed.

Our space does not permit us to follow the author in his descriptions of the various circumstances which may give rise to slight modifications in the operation, and indeed it is scarcely necessary to do so, as no two cases exactly resemble each other, and any surgeon who was thoroughly acquainted with the general principles of the operation would be able to adapt slight changes to the nature of the case. It is well, however, to know that Dieffenbach has succeeded in forming satisfactory noses from the hairy scalp, the connecting bridge being a long narrow strip of the skin of the forehead. The advantages of this method are that very little scar of the forehead remains; the scalp is so firm that it does not contract, and thus a smaller flap is necessary, and the nose remains firmer. The hair is prevented from growing by solution of bichloride of mercury. Without stating anything, however, against this method, the author says he only practices it when the other operations are impracticable.

*Partial rhinoplastic operations,* or restoration of a part of the defective nose, is effected by transplantation either of the skin of the forehead, or of that of the eyelids, cheeks, upper lip, or of the nose itself. The chief difficulty is in adapting the flap exactly to suit the various forms of defect, and also from the want of correspondence between the new and the old parts. When the ridge of the nose is entirely absent, a furrow occupying its place from destruction of bone, the edges are pared and a proper sized flap cut from the forehead and adapted as before. When the upper part only of the ridge is defective, the point remaining, the same directions will apply. When the bony part of the nose is covered with sound skin, the alæ alone being wanting, the forehead-flap must be as broad as in the operation for total restoration, and the septum must be also of the same breadth. The connecting bridge must of course be very long. The model is cut, fixed to the forehead, incisions made and flap separated, and then the bridge is to be carefully separated in the neighbourhood of the corner of the left eye; the union of the wound of the forehead, and adaptation of the flap as before described. When the alæ have been drawn inwards by cicatrices, they may be raised after division of the latter, and afford great assistance in the formation of the nose, as, instead of adapting the forehead-flap to the furrow made in the face by incision, it is merely necessary to pare the edges of the alæ and bring them into apposition with the edges of the flap. When the septum alone is defective, it may be restored by transplanting a long flap, the breadth of a finger, from the centre of the forehead, or it may be formed from the upper lip. As the latter operation is not described in our former articles, we extract the remarks of our author:

"*First method.* Flap taken directly below, and mucous membrane turned outwards. The septum is entirely wanting. The middle of the upper lip is twice cut through, and the small isolated strip, from which the red portion of lip is removed, is united by insect needles with the edges of the wound of the point of the nose. Von Ammon, Liston, and Fricke operate in this way. This method has the great advantage that the flap undergoes no twisting, as it is carried directly upwards. I can prove the great value of this method from personal observation. The everted mucous membrane becomes dry, and assumes the nature of epidermis.

"*Second method.* Flap taken from below and turned. A strip of skin is cut from the whole thickness of the lip. The incision on the right side divides the right nostril in order to facilitate the turning of the flap; this is then loosened from the bones, turned back, and united by means of three insect needles with the wounded point of the nose, after removal of the red edge. The division of the lip is to be previously united by three strong insect needles. . . .

"When the cartilaginous septum remains, the external part alone being absent, I first pare away the edges, and wound the point of the nose; then I cut a perpendicular strip from the corium of the upper lip, and fasten the upper border, the red edge being previously removed, to the point of the nose with insect needles. This method generally succeeds perfectly.

"When large strong cicatrices existed on the lip I have excised them, and formed a septum from the substance of the cicatrix, which restored the form perfectly.

"*Third method.* Septum formed by oblique divisions of the upper lip. In cases of thin long upper lip and small orifice of the mouth, I have endeavoured to avoid further lessening of the mouth by taking the strip of skin in an oblique diverging direction beneath the nostril, loosening it from its attachment, turning and uniting with the point of the nose by sutures." (pp. 345-7.)

Death of the flap is apt to occur in this operation unless it have been so well loosened that the bridge is not much pressed in twisting. It is better even to leave a little gaping at first, and complete perfect union afterwards by division of the bridge.

"*Fourth method.* Immediate union of the point of the nose with the upper lip. I make two perpendicular descending incisions through the whole thickness of the lip, from the two nostrils to the middle of the upper lip, and at the highest point. These are united by a transverse incision. The upper border of the wound is fixed with forceps, the skin loosened and carried forward as the bridge; then the anterior and inferior border of the point of the nose is wounded, and the flap being drawn upwards, is fastened to the nose by three insect needles. No union of the wounds of the upper lip should be attempted." (p. 347.)

We have given a literal translation of the description of this fourth method, but must confess that we do not perfectly comprehend it. How deep is the upper transverse incision to be? If the two perpendicular incisions commence immediately beneath the nostril, and the transverse incision is at their highest point, what bridge can there be above the upper border of the wound? Conciseness of description is always to be recommended, but it may become a fault; and when carried too far, needlessly puzzle the student.

The septum may also be formed from the ridge either of the old or new nose, bringing down a flap cut from the ridge, and uniting it, after turning, with the point of the nose and the upper lip by needles, and uniting the edges of the wound of the ridge after removal of the flap. This operation is described in our Seventh Volume, p. 403.

Some interesting observations follow *On the means of closing openings in the bony structure of the nose.* In these, as in all the other plastic operations of Dieffenbach, he states he has given not studies upon the dead body, but "real portraits for which living men have sat."

1. Suppose the opening in the nasal bones is of the size of a large pea, the surrounding skin thick, healthy, and inverted. At the upper and lower part of the opening a small wedge is cut out to give the opening the form of a pointed oval, an incision is made around the edges of the opening, and the outer borders separated from the bone all round to the extent of five or six lines; they may then be brought together and united by insect needles and twisted suture.

2. If necessary, incisions may be made at the side of the opening after paring the edges; this facilitates their approximation, and after their union the healing of the side-wounds is effected, scarcely any scar remaining.

3. In case of a large hole in the upper part of the root, of the circumference of a bean, with the edges towards the corner of the eye, and surrounded upwards and outwards by an elevated swollen margin, the thin part of the border is to be surrounded by a semicircular incision; then the opposite border is also surrounded by a convex incision, which runs outwards about four lines from the edge of the opening. The bone of the flap thus formed is separated, so that it may be easily turned over the opening, and its convex border united by the twisted suture with the concave one on the other side of the opening.

4. A somewhat larger opening in the same situation may be closed by transplantation from the forehead. At some little distance from the opening it is surrounded by an incision, and the ring removed only when it is diseased. The skin around is loosened, and a flap, the shape of which is determined by a plaster model, cut from the lower part of the forehead, and adapted to the wound around the opening.

5. The skin of the cheek should only be taken to close these openings when they are in its immediate vicinity; otherwise it is better to take the flap from the forehead. After the incisions of the edges, a round flap is taken from the cheek; a little external to the ala nasi is the most convenient spot; the flap is fixed and the edges of the wound of the cheek approximated, by drawing forwards the skin of the side of the face.

6. In some very rare cases, as when a very large opening exists close to the eyelid, and this is drawn towards the opening by cicatrices, the skin of the eyelid may be used as the flap, and at the same time ectropion consequent upon its traction towards the opening be removed.

7. The flap may be formed from the substance of the lip. A large hole may exist in the under part of the root of the nose, and beneath the edge of the orbit, so that the internal structure of the nose may be seen. Cicatrization has drawn the upper lip, and often the whole corner of the mouth into the opening, the mouth being thus nearly doubled in length. This must be rectified before closing the nasal defect, in order not to disfigure the mouth more for the benefit of the nose. This operation is described in successive steps by division of both lips, their reunion forming a new angle of the mouth, and then the remaining part of the upper lip used as a flap for closing the opening in the nose; but the wording is so careless and contradictory, that we have in vain endeavoured to comprehend exactly how the operator is desired to proceed.

*The elevation of a nose* which has sunk in may be attempted when the bony parts have been destroyed, without injury to the soft parts, on their inner surface. The latter must not be drawn completely inwards, nor their under support be entirely wanting, or the nose would resink after its elevation. The operation is commenced by dividing the nose throughout its whole length by four incisions. Two of these incisions, which commence at the internal corner of the eyes, and pass to the posterior inferior border of the alæ, divide the skin and cartilage at the limit between the side of the nose and the cheek. The other incisions have the ridge of the nose between them; the point of the septum remains hanging together. When the base of the nose has been loosened from the bones, the inner border of the edges of the upper incisions are to be slightly pared, and the outer borders of the under, to assist in the proper replacement of the nose. The two upper incisions are then united by the twisted, and the two under by the interrupted suture, two tubes being placed in the nostrils. The neighbouring skin of the cheek is then, by means of two long needles carried across it and two small leather straps, pressed together, and the nose thus more pressed forward. The sutures are afterwards removed, the inner covering of the nose cauterized for a long time, and elastic tubes worn until the nose has no tendency to sink.

In cases of *flattening of the nose* after total destruction of the bones, one or more flaps may be united one over the other at different times.

When flattening occurs from relaxation of the skin surrounding the base of the nose, portions may be removed, and the reunion of the edges remedies the defect.

Elevation of the flattened ridge may also be effected by making an incision from between the eyebrows to the point of the nose in a direct line, loosening the edges, and bringing a flap half an inch broad, cut from the middle of the forehead, which is united by needles with the edges of the nasal wound. The flap must be well supported underneath, or it will sink inwards.

A singular method of elevating the whole nose is described as very useful and often practised. Two small splints of stiff leather are placed at each side of the nose, and these, with the nose also, are transfixed by long thick insect needles. The heads of these on the left side are drawn to the splint, and on the right the points are cut off to leave about a quarter of an inch of the needle projecting. These are then surrounded by threads to prevent their escape, and press the splints together. After some days, when the punctures suppurate, the needles are bent to the shape of a ring, and being fixed by pincers, the nose is thus pressed more outwards: afterwards the needles are still more bent. When they have remained two or three weeks and produced much surrounding swelling, the needles are removed easily by clipping off their heads. Then the skin is cleaned, new splints applied, and the nose transfixed in two or three other spots. In the mean time the first punctures heal, and their orifices are drawn a little inwards, and the nose is held together by the contraction of the thickened cellular tissue, into which their canal is converted.

Small defects of the point of the nose, and separation of the integumental portion of the septum, may be easily remedied by paring the edges, loosening the surrounding skin, and then bringing the edges together and uniting by twisted suture.

*Defects of the alæ nasi* are very common, particularly in young persons. When slight, paring and subsequent approximation of the edges of the cleft or fissure, is all that is necessary. If the symmetry be thus destroyed, a similar operation may be performed on the sound side; and then if the ridge be rendered too prominent, a small portion of the cartilage may be removed where it unites with the integumental portion of the septum.

Some modifications of this operation, to suit particular cases, are described, but our space will be better occupied by more important matter.

Various *deformities of the nose* may be removed by subcutaneous operation, as division of cicatrices, which draw the nose inwards; this is easily done by a small scalpel inserted up the nostril. Depressions of the cartilaginous ridge of the nose are also treated by inserting the knife into the nostril, loosening the skin around the depression, and dividing the cartilage transversely with a tendon-knife, filling the space up afterwards with charpie. Depressions of the alæ also may often be remedied by similar or by crucial incision of the cartilage, without injuring the external skin, keeping the nostril stuffed with charpie until the parts have healed. When the nose is crooked from obliquity of the septum, the deformity may be remedied by the removal of a long oval portion of the cartilage of the overhanging part of the septum. When the obliquity of the nose is congenital, or arises from wounds or disease, it is remedied by raising the skin of the ridge at the point between the bones and cartilage, transfixing it, and with a small concave knife dividing both cartilages quite down to the cheek, where they join the bone and the cartilaginous septum also—of course beneath the skin. Charpie is then placed within the nose, and the nose is drawn to the side necessary to straighten it by means of straps of plaster passing from the temple over the nose to the lower jaw. This is to remain long undisturbed, and replaced in the same manner after removal. Generally the healing and removal of the deformity are rapid, and the author has never seen any ill effect follow this operation. It certainly appears to be a simple method of removing a great personal defect.

*The after-treatment.* Generally speaking a local and constitutional antiphlogistic treatment is now found most successful after plastic operations; contrary to the formerly received opinion, that a stimulating treatment was necessary to elevate the diminished vitality of the part. This is only necessary in weak persons, when the parts remain flaccid, cold, and deficient in blood; warm aromatic applications and a covering of flannel may then be used to sustain the heat of the parts. More often a congestion of the flap leads to its death, the blood entering more easily than it can escape. Cold applications must then be used, and leeches applied upon the bridge, the surrounding parts, and even the flap itself. The bleeding is encouraged by fomentations, and sometimes a small portion of the edge of the wound is removed, and free bleeding generally follows. General bleeding is advisable if there be much constitutional excitement. If nevertheless gangrene should follow, stimulating and aromatic applications must be used, granulation of the inner surface of the nose encouraged; and when the healing is completed, the defect must be again remedied after the methods before described.

Some *after-operation* is almost always required, even when the first has been most successful, in order to perfect the produce of art. Small prominences must be pared off; small depressions elevated; puffiness removed by superficial cauterization; central elevation of the flap lowered by excision of a small flap from its highest part; the connecting bridge, as soon as the nutrition of the flap is secured, is to be excised by two elliptical incisions, and the edges united by fine insect needles. In this manner all bridges of transplanted flaps are to be excised, unless the portion of skin is required to cover some other defect.

An operation is described as the favorite of the author, in lengthening and improving the form of new noses, by making two diverging incisions from the root, where both commence, along the sides of the ridge. The long tongue-shaped wedge thus formed is loosened from above, so that it only remains attached below. The skin of the side of the nose is then loosened, and the edges brought together over the ridge. The wedge is pressed downwards to the point of the nose, which is then long pressed outwards, and the wedge united with sutures.

The frontal cicatrix is best thickened or condensed by the application of lead lotion and a compress. Ointments thin the epidermis, and do harm. It is often advisable to lessen the cicatrix by excision of long portions of the shape of a myrtle leaf, or entirely to extirpate it, only taking away so much at a time that the edges can be reunited. "In young girls I have generally removed it completely by repeated operations, so that no trace of any operation having been performed on their forehead could be discovered." (p. 372.)

Dieffenbach has only once attempted the support of a sunken nose by a metallic plate, and in that case without permanent success, having been obliged to remove it, and transplant a flap from the forehead.

As we have already, (vol. VII, p. 390-3,) when commenting on the operations of Taliacotius, and Von Gräfe, described in general terms the method of restoring the nose by a flap taken from the arm, we do not think it necessary to enlarge here upon this operation; but it may be interesting to know the opinion of the author, after his enlarged experience, upon the relative value of the two modes of forming the flap, the one from the forehead, the other from the arm. He says, each have their advantages and disadvantages, but generally the former is far preferable, and the latter only to be adopted when the former is impracticable.

The advantages of the forehead flap are, that it is firmer and thicker, and the nose consequently is less liable to shrivel—the greater proximity of the forehead renders the union of the flap more certain—the operation is more easily performed—the patient remains much more comfortable during the healing process—and the healing is more rapid. As a *per contra* it must be allowed that the cicatrix on the forehead is a disadvantage, and after the removal of this, the alteration in the countenance produced by the approximation of the eyebrows. Some danger also may be apprehended from the denudation of the forehead when a very large flap is required.

The advantages of the other operation are, that the cicatrix of the forehead is avoided—the countenance is unaltered—and its applicability when the skin of the forehead is diseased. The disadvantages are, the greater difficulty of its performance—the tormenting situation of the patient after the operation, during the progress of union between the arm and face—the annoyance of pus flowing over the face, and drying by evaporation, when the flap is applied immediately after its elevation from the arm—the limitation of the method to the restoration of the anterior part of the nose—the diversity in colour and thickness between the skin of the face and that of the arm—the frequent failure in the union of the flap—and, lastly, the diminution and withering of the nose from resorption of the subcutaneous layer of fat.

The chapter on nasal plastic operation is excluded by a detail of the case we have noticed, (vol. XIX, p. 399-400,) and some anecdotes descriptive of the joy and gratitude of patients who have been restored to their friends and society after years of solitude and distress. One, some years after the operation, wrote, "I have lost the whole of my large fortune, but I am happy, *for I have a nose!*" Lavater says, a beautiful nose is worth a kingdom, and probably no one knows the value of his own until he has lost it.

The thirtieth chapter is occupied by the methods of *dilating and perforating the nostrils,* when they are closed or adherent. The evil may be congenital, or the result of disease, and may be present in natural as well as artificial noses. Simple or conical incision; or removal of the cicatrix or other substance closing the nostril by excision; the application of caustic; and wearing metallic or elastic tubes, are all that is required.

THE EAR. Otoplastic operations are described in chapter 31. Complicated wounds or lacerations of the external ear are common. The edges are to be equalized if fresh, and pared if they have become covered with cuticle, brought into apposition and fixed by suture. The replacement of an entire ear cannot be effected, the new ear remaining in a formless mass, but parts are successfully restored as the upper ear, the helix, and the lobule.

The edges of the defective part are pared, a flap of the requisite size raised from neighbouring skin, and fastened by suture, oiled lint being placed beneath the flap behind the ear. When the flap is united, and cicatrization of the edges commences, a rather larger strip of skin than the defect requires is cut from the skin of the head, which gives a round form to the border. Interposed lint prevents adhesion.

When the restoration of the lobule has been required, it has generally been lost by burning, and the remains united to the cheek by a common cicatrix. The method by sliding or displacement is here practised—a descending incision is made from the free border of the ear, and then a second on the opposite side. The two inclose between them a portion of skin, one third broader than the healthy lobule, and are united below by a curved incision of the form of the lobule, and then the flap thus formed is separated from its under attachments. The healing of the wound is effected by loosening the edges, and bringing them together, and this is facilitated by cutting out small pieces of integument, so as to make the ends of the wound pointed. The wound heals by the first intention, and the flap becomes covered by cuticle. Small irregularities are removed by superficial paring, and the lobule generally proves to be a good one.

THE MOUTH. We are now introduced to the operations *Chiloplastik* and *Stomatopöesis,* or those performed for the restoration of the lips and mouth when destroyed, or the removal of their deformities. The general remarks on the union of the wounded lips are worthy of attention, and we extract them accordingly.

"In no wounded part of the body is the twisted suture so necessary as in wounds of the lips, where it especially offers three great advantages, 1st, It brings the edges of the wound and the substance between the needles also into close contact;—2dly, it suppresses the most violent bleeding;—3dly, it so fixes the parts during the healing process, that eating and speaking do not do much harm. The interrupted suture, on the contrary, allows after-bleeding, between the edges, and swelling and separation from each other of the interstitical substances, and does not fix the lips.

"The union is effected by insect needles and thick cotton threads; the larger the wound, the stronger the binding; when there is great loss of substance, the longer and stronger must be the needles, in order that in their application they may reach from one wounded border to the other. Here they must be rather thick, in order not to bend from the tension of the edges. The needles are applied during the most violent bleeding from the edges, as a means of suppressing it; whereas frequently in other situations it is necessary to wait until the wound bleeds no longer. The coronary artery is never to be tied, because the ligature prevents union, and an insect needle and twisted suture, applied to the spot, instantly suppress the bleeding." (p. 398-9.)

The needles are passed three or four lines from the edges of the wound, and close to the mucous membrane. We have described this little operation at length, as the twisted suture, in many of its applications, is regarded by Dieffenbach as a *specialité* of his.

In the following chapter, the 33d, we find the operation for *Harelip.*

Our author has operated upon a *thousand* cases, and says that while union has taken place at every age, from a few days after birth into extreme old age, it is better to wait until dentition is accomplished, as when performed very early, the cicatrix is apt to yield as growth advances. It is not necessary to follow the details of this operation, and its modifications to the different forms of harelip, as we find nothing not generally known and practised here. Dieffenbach prefers the scissors to the scalpel for paring the edges, and uses much thinner needles than are in fashion here, as he says failure frequently follows the inflammation and suppuration set up by thick needles. Instead of the waxed silk often employed, he uses thick soft cotton thread, as he says the former frequently causes the needles to break through the skin, and leave ugly cicatrices, like pock marks, upon the lip.

The operation for *ectropium of the lips* is one of the most difficult, but successful, of plastic surgery. The defect is always connected with contraction of the external skin, and generally is the result of burns; sometimes of severe herpes.

In a simple case of ectropium of the under lip, where a small portion only of the lip is turned downwards, the operation resembles that of cancer of the lip, a wedge being cut from its middle, with the point directed to the chin, loosening of the edges of the wound, and application of needles. The external skin is of course left uninjured here, (although the author does not say so,) the wedge only including the mucous membrane and submucous tissues.

In the more advanced stage the lip is no longer a screen covered by skin and mucous membrane, but merely a surface of mucous membrane, drawn down to the chin, and covering it like a "red half moon," leaving the teeth and under jaw exposed. By incisions from one corner of the mouth to the other, which descend to the chin, and loosening from the bones, a portion of the everted lip is extirpated of a rounded-triangular shape, like the lower half of *the spade* of cards cut across. Then from the two upper corners horizontal incisions are made and the sides loosened. They are then brought together, and united by insect needles. If the surrounding skin be very firm, lateral incisions require to be made to the edge of the jaw, to facilitate the sliding or displacement of the integument.

Sometimes, by cicatrices extending from the breast, the lip is everted, and brought below the point of the chin. In such a case, Dieffenbach has operated successfully as follows:—

"First, the cutting of a half-moon shaped flap from the red skin. Commencement and termination at the angles of the upper lip. Loosening and turning up of this flap. Excision of two small wedges from the present corner of the mouth, and union by sutures. Carrying over of a cross slip of plaster, by which the outer surface of the lip flap is pressed against the inner.

"Then two narrow incisions through the hard cord-like cicatrix, from without downwards to the sternum. Loosening of the borders. Bending of the head backwards. Direct approximation of the edges is thus effected. This pyramid, cut from the cicatrix, moves upwards, and is fastened to the side edges, and the inferior point of the wound can also occasionally be directly closed. Strips are afterwards taken from the centre of the pyramid, and the edges united, and thus greater elevation obtained." (p. 414.)

We have given this description in the author's own words, because the operation appears to be a valuable one, and also because the directions are so concise that some misconception might easily take place.

Ectropium of the upper lip arises from similar causes, and operations upon similar principles remove it.

When the corner of the mouth is turned outwards, a wedge of the mucous membrane is excised, the base at the corner of the mouth, the apex towards the cheek, and the edges are united by twisted suture.

In a case of total ectropium of both lips and both corners of the mouth, the operation just described is performed upon the corners, and then the upper and lower lips are replaced by the usual methods.

The double lip is remedied by excision of the false inner lip, with fine scissors, and union of the wound by a number of small sutures.

In our Nineteenth Volume, (p. 404-8,) and Seventh, (p. 407-8,) we have entered at some length upon the subject of operations for the restoration of both upper and lower lips, in the former commenting particularly upon the methods of M. Serre, and in the latter upon those of Dieffenbach himself. It is not necessary, therefore, to retrace our steps, especially as our author does not appear to have modified his former proceedings.

For a description of the means of enlarging a contracted mouth, we can also refer to vol. VIII, p. 409-10, and vol. IX, p. 402-3. Since his former publication, the author has repeatedly performed this, *his own* operation, with success, and upon persons from the most distant countries.

THE CHEEK. The thirty-seventh chapter contains the meloplastic operation, or that by which a greater or less portion of lost cheek is renewed. We must again refer to the interesting case of Dr. Mütter, detailed vol. XIX, p. 403-4. In vol. VII, p. 410, will be also found a notice of Roux's operation. It is necessary, however, to recur to this subject.

Dieffenbach observes that the generality of so-called meloplastic operations which have been recorded, do not deserve this title, being merely restoration of a part of the cheek by displacement of surrounding skin, or closure of openings between the cheek and nose, or in the nose itself. He says—

"When the middle part of the cheek is defective, the mouth is generally drawn obliquely outwards to double its length, and its corner is close to the malar bone. Here, as described when treating on the restoration of the lips, both lips are divided perpendicularly at the spot where the corner of the mouth is to be made; the wounded edges of the proper mouth are brought together by sutures, the displaced part is loosened round about from the bones, the mucous membrane taken away in order to obtain bleeding edges, and insect needles applied to effect union.

"When the defect of the cheek is of greater extent, but the corner of the mouth less drawn, the mouth is first restored, and its corner made smaller by excision of the borders. Then the borders of the defect are excised, and stitched with thick needles and thread; at the border of the nose a deep incision is made, and a second, in the form of a half-moon, on the opposite side, both being of such a depth that the middle can be united.

"When the corner of the mouth is drawn upwards into a great defect, and the under eyelid is drawn downwards as a total ectropium, I have made use of the superfluous part of the lip to close the under part of the defect; I have obtained the covering for the middle part of the lost substance, by freely loosening the borders of the cheek, and stretching them greatly after the lateral incisions, and several times I have turned the whole everted lower eyelid, covered at the time with healthy epidermis, into a wedge-shaped flap, have brought this down, and so completely replaced the lost substance. The eyelid was then restored by integument from the temple.

"In other cases where the defect was nearer to the nose, and where three fingers could be passed through it into the mouth, when the nose was large, and the defective ala bounded the opening, I have loosened broad flaps from the whole side of the nose, and have obtained substance for the most external part by sliding the flaps, or by lateral incisions, and have united the nose and cheek flaps by the twisted suture, and completely replaced the nasal defect from the forehead.

"I have frequently closed large openings, particularly in the central part of the cheek, by circular incisions at a considerable distance from the borders of the opening and loosening of the flaps, which commence by obtuse points, and terminate in a broad base. These strips are bound spirally with each other, and with the surrounding parts, upon the surface, in the form of a small circle." (pp. 431-2.)

The author has transplanted the soft parts from beneath the lower jaw and chin, to restore these defects, but has never used the skin of the neck for this purpose, as the danger of denuding the neck is too great, and the distance is as great as from the forehead. The integument of the arm could be used, but only when it could not be elsewhere obtained.

Most of these operations require to be performed in successive steps, and often some months must be allowed to intervene between each operation, and no new one attempted until cicatrization is perfectly accomplished. This is especially the case in the extreme deformities following malignant ulceration, where not only the half of both lips, but the *ala nasi,* on the affected side, are destroyed, with the whole cheek. The alveoli are also wanting, so that the tongue is freely exposed. The border is thin, and adherent to the bones; and often ectropium of the under eyelid is also present, the jaw being also fixed by contraction of the cicatrices, and of the masseters, or perhaps from true anchylosis. In such cases, by repeated operations, after months or years, first freeing the jaw by division of the masseters, &c., and then replacing part by part, after the methods we have described, successful results have been obtained.

THE PALATE. We now come to the 36th chapter, on the palatine suture, (Gaumennaht,) but after the lengthened notice we gave in our former article on the operations of Mr. Mütter and Mr. Fergusson, (vol. XIX, pp. 412-16,) it is not necessary to return at any length to this subject. We would, however, remind our readers of the valuable paper lately read at the Medico-Chirurgical Society, by Mr. Nasmyth, on the mechanical means of relieving palatine fissures. In some cases the mechanist can do more than the surgeon, in others the reverse holds good, while each might assist the other more frequently than they do.

In cases of small holes, or openings in both the soft and hard palate, Dieffenbach employs with great success a concentrated tincture of cantharides, with which the borders of the opening are pencilled several times daily. Inflammation and granulation of the edges are followed by union, while if the potassa pura be used, a portion of substance is lost, and the granulation is not sufficient to close the opening, which remains larger than before. Larger openings are, of course, treated by paring the edges, and union by suture.

In closing fissures of the soft palate, leaden wire is said to be far preferable to silk, and much more easily applied. It can be drawn sufficiently tight to keep the wounded edges close together, while silk, if so drawn, would cut through those delicate textures.

Adhesion of the velum palati to the posterior wall of the pharynx, causes great suffering from stopping the communication between the nares and air-passages, deafness from closure of the eustachian tube, &c., and therefore, although the operation is very difficult, the adhesions must be freed by means of a long scalpel, making a transverse incision, about half an inch below the adherent border of the velum. The edge is fixed by a hook, and drawn from the wall of the pharynx. Then a lancet-formed knife, the flat surface of which is curved, is used and directed upwards, to loosen the velum, the separation of which is completed by scissors, also curved upon their flat surface. The upper adhesions are destroyed by passing a blunt curved iron instrument, like a very small spatula, along the inferior nares. This operation would be rendered unsuccessful by a fresh adhesion of the parts together, unless sutures were applied. A ligature is prepared, with a small curved needle at each end, and with one of the needles the velum is transfixed a few lines from its edge, and the needle brought out at a high point, on the anterior surface of the palate. The other needle is used in the same manner, the ligature being passed a short distance from the side of the other. Then the ends of the thread are tied together, taking care that the edge of the velum is left about half an inch distant from the palate.

As we have referred to Mr. Nasmyth's paper on the mechanical treatment of palatine fissure, it may be well to state that Dieffenbach considers that, in general, all mechanical means for closing openings, or fissures of the velum are not only useless, but injurious and dangerous. With regard to those of the hard palate, wearing anything between the edges of the opening, gives relief for the time, but generally causes enlargement of the opening, so that if the size, or other circumstances, render an operation unadvisable, it is better to cover the palate with a gold plate fixed to the teeth. Of course this would be left to the dentist, but if no such person be in the neighbourhood, any mechanic could make such a plate if the surgeon took a model of the palate in soft wax, harden this in cold water, and upon this make a cast in sulphur, or plaster of Paris. The gold plate, formed upon this cast, would form the artificial palate, and be fixed by gold wire around the back teeth.

In cases of holes in the palate, the edges of which are so callous that an operation would be unsuccessful or impossible, the opening may be stopped by wearing a double piece of Indian rubber, without the danger of its enlargement. Two pieces of Indian rubber, of the thickness of thin pasteboard, are cut about four or five lines larger than the opening, and between them, in the middle, a small round piece of the same thickness is laid, and these three layers are transfixed, and sewed together with waxed thread. One plate thus is made to lie on the anterior, the other on the posterior side of the palate, and the small middle strip in the openings, with the edges of which it is not in contact, as it is smaller than the opening. When the patient wishes to insert this obturator, he softens it in warm water, squeezes its layers together with a pair of forceps, and passes it through the opening, standing with widely open mouth before a looking-glass. It is removed once a week to clean it, or to apply a new one. When the opening is so small that some hope of a closure remains, the edges should be pencilled with tincture of cantharides. We have lately applied this elastic obturator with most satisfactory result upon a gentleman who had undergone three unsuccessful operations, by paring the edges and applying sutures. We took a model of the palate in wax, and upon this made a plaster cast. This saves a great deal of unpleasant manipulation in the patient's mouth, and an exact fit may be easily obtained. Our friend was quite delighted with the result, and his wife amuses herself by making the plugs, so that with the cast they are quite independent. It is really surprising how much happiness may be conferred upon a family by so simple a contrivance. How, then, can a man practise with a clear conscience who does not keep himself on a par with the knowledge of his age? We think it is Dr. Baillie who says, "In other professions ignorance may be *folly;* in ours it is *crime.*"

EYELIDS. The blepharoplastic operations, or those upon the eyelids, occupy the following chapter. In our Fourth Volume, (p. 483,) Von Ammon's operation is described and illustrated by a woodcut. Some of Dieffenbach's operations, particularly for the cure of ectropium, are referred to in vol. VII, pp. 404-7, and also illustrated by cuts. There are, however, some points in his present volume which require further notice.

These are the most difficult of the operations of plastic surgery. A nose may be considered a good one when it is like a nose, although it be too large or too small—a mouth is a good one when the lips cover the teeth and the saliva does not escape; but the eyelid must be exactly like the lost one: it must be moveable, and close the opening between the lids and cover the eyeball, when the operation is considered to be of any value.

In *wounds of the eyelids* Dieffenbach insists strongly on the advantages of his fine insect needles in producing union by the first intention. A sufficient number must be applied to effect exact apposition. If the edges do not correspond, they must be made to do so by the use of fine scissors. If a large piece of skin be lost, and the edges cannot be united, subsequent ectropium is avoided by making an incision a quarter of an inch from the edge of the wound, which allows the edges then to be united. Opening of the lids during the healing process to be prevented by a strap of plaster, carried over them both. About the third or fourth day the needles are to be carefully removed, and the adherent wound supported by strips of plaster, to prevent reopening. If the needles are properly applied, no scar remains.

The operation for coloboma—a perpendicular cleft in the eyelids, with hard edges—is the harelip operation in miniature. Exact approximation of the edges by careful application of the needles insures the cure. Cold lotions must be used after the operation.

*Tarsorhaphie,* or the operation to diminish the size of the opening between the eyelids, is required when this is enlarged by surrounding cicatrices. Von Walther first proposed it. It consists in the union of the unnaturally elongated part of the opening, the edges of which are fixed by hooked forceps, and cut away to the breadth of half a line with a small scalpel, the two incisions meeting over the commissure towards the temple. Union is effected by insect needles. The most anterior suture, the nearest to the eyeball, should be a fine stitch. If cicatrices have caused the disease, they must be first removed, as before directed, and then tarsorrhaphie performed.

*Lagophthalmus,* or hare's eye, is a condition in which the eyelids cannot be perfectly closed, so that a part of the eyeball is seen through the opening. If produced by small superficial cicatrices, these must be excised, and the edges of the wound left united, as before directed. When the cicatrices are transverse, he employs subcutaneous division in several spots of the whole upper lid and its cartilage, which is then drawn down and fixed during healing by strips of plaster. A long hard perpendicular cicatrix which shortens the middle of the lid is excised by long elliptical incisions (), and the union by needles draws the lid down. Division of the levator palpebræ is sometimes required.

*Blepharoptosis,* or falling of the upper eyelid, depends upon a loss of balance between the actions of the orbicularis palpebrarum and the levator palpebræ superioris. It is removed either by a subcutaneous division of the orbicularis, or by shortening the depending lid by the excision of a horizontal portion of its integument. The wound may be united by sutures, or, if the skin be very lax, by granulation.

*Ankyloblepharon,* or the union of the edges of the lids with each other, and *Symblepharon,* or the union of the lids with the eyeball, or a complication of the two may be congenital, or the result of inflammation. We need not describe the mode of dividing these adhesions; the operation in the first case is generally successful, in the second only when a part of the ball is adherent, and the success in both cases depends principally upon preventing subsequent reunion.

We find nothing new with regard to entropium, and as we have before, as just stated, given the author's operation for ectropium, we pass to the *blepharoplastic operations proper,* and extract the author's present mode of forming the under eyelid from the integument of the temple:

"1. *Wounding of the base.* First a semicircular incision is made between the defect and the eyeball, through the free conjunctiva; the edge is fixed by fine hooked forceps, and loosened as far as the fold (or spot where it is reflected). Then a small pointed scalpel is inserted at the internal canthus, and carried obliquely downwards until it reaches below the malar bone. A perfectly similar incision is made from the external canthus to the same point. The flap of skin thus isolated has the form of an inverted pyramid, the base of which is beneath the eyeball. The upper edge is fixed by the forceps, smoothly separated (abpräparirt) from above downwards, and afterwards carefully applied. If the inferior portion of the orbicular muscle is not destroyed, as in ectropium, it is to be carefully preserved.

"2. *Formation of the flap from the temple.* The skin close to the external commissure is divided, and the incision carried almost horizontally, but at the same time somewhat obliquely downwards to the temple, so that the flap above may be a fourth part wider than the upper part of the defect; the knife is then brought obliquely downwards, approaching somewhat more to the border of the defect. The incision ends below, opposite the point of the pyramid. Then the flap is loosened from above to below (holding it with forceps) by flat and even strokes of the knife, so that no inequalities may be made. As soon as the bleeding has perfectly ceased we commence with—

"3. *The laying down and stitching of the flap.* This is pushed from its situation to its new seat, and the pyramidical wound covered with it; the whole anterior border of the flap is then united from below upwards, with the wounded border of the facial integument, by a considerable number of insect needles and twisted suture, the flap being thus somewhat drawn up, so that its upper border may jut outwards: the highest corner must be exactly united by a stitch with the internal canthus.

"4. *Fastening the conjunctiva.* The halfmoon-shaped cut border of the conjunctiva is then so united with the upper border of the flap by a quantity of fine interrupted sutures, that the knots may lie upon the outer skin." (p. 496-7.)

The dressing consists in filling the side wound with fine charpie, and then carrying long slips of plaster over the side of the face to the opposite side of face and head, preventing stress upon the sutures by previously applying to each side of the united wounds fine linen, folded six times into a long compress, a few lines broad. Antiphlogistic after-treatment. The dressing very carefully removed, commencing about the third or fourth day by removing here and there a needle which presses, or is surrounded by redness, or where the union is complete. The next day all is removed, and fresh strips of plaster applied. The conjunctiva sutures must often be removed before the others.

Elevation of the flap, which occurs a few days after the operation is performed, is counteracted by the combined cicatrization of the wounds of the temple and face, the contraction flattening the flap. If the orbicularis remained intact, the lid is moveable, and the eyelashes alone are wanting.

The upper eyelid is formed precisely in the same way as the under, carefully avoiding the supra orbital nerve.

Partial is very much more simple than total restoration. It is the simplest application of the operation, by sliding or displacement.

*Transplantation of the eyelashes.* It has been established by experiment that strong hairs, freshly plucked, take root when they are inserted into small oblique punctures, and protected by slips of plaster. The hair must be strong and young, not just about to fall away. Grayness of the hair is of no consequence, as it is the age of the hair, not of the individual, that is to be considered. Dzondi was the first to apply these physiological facts in practical surgery, planting a row of ciliæ upon an artificial eyelid. Dieffenbach does not appear to have followed his example, but he says, to increase the satisfaction of the patient in a case of successful blepharoplasty, the necessary quantity of ciliæ might be plucked from the other eye by forceps, and inserted in small oblique punctures, half a line in depth, made along the border of the lid, the parts being then covered by fine strips of plaster.

*Lachrymal fistulæ.* Here is the title of the 48th chapter:

"DACRYOCYSTOSYRINGOKATAKLEISIS"

or healing lachrymal fistulæ by transplantation! This operation simply consists in paring the edges of the fistula, loosening the borders, and assisting the necessary integumental displacement by lateral incisions. Insect needles and twisted suture of course complete the operation. We have seen many severe and obstinate cases of lachrymal fistulæ, but never one which did not close soon after the patency of the nasal duct was re-established. Cauterization or simple paring of the indented edges of the facial opening is occasionally required, but when once the natural passage of the tears is established, little else is necessary, as spontaneous closure invariably follows. This leads us to make a few observations on the means of reopening the nasal duct, as nothing can be more unscientific than the proceedings still adopted by the vast majority, both of English and continental surgeons. What is the disease? Simply a stricture or closure of the nasal duct, causing a retention of tears in the lachrymal sac. The consequences are, the flowing of tears over the face, with subsequent irritation and excoriation; or inflammation and suppuration of the sac, and an opening on the face, through which the pus and tears escape. Now, putting aside palliative measures, the only means of curing either of these conditions is by keeping the nasal duct freely and permanently open, and nineteen out of twenty surgeons, at least, do this by passing a style through the sac into the duct; to do this, making an opening in the face, if the disease had not done so. The patient is obliged to wear the instrument constantly, and a most unsightly thing it is on the face of a lady. The whole process is unscientific and unsurgical, not to say barbarous. Let us compare the case with one of stricture of urethra, causing retention of urine. Would any man in his senses relieve such a case by cutting through the perineum as long as there was any possibility of opening the natural passage? And if perineal abscess and fistula had formed, would he attempt to remove the stricture of the urethra which had produced it, by making the patient wear a style in the perineum? Yet this is done every day on the face, with the additional disadvantage of the style being visible, and an annoying disfigurement. Any one with a little practice can open the nasal duct through the natural orifice, as easily as he can pass a catheter along the urethra. The profession is under great obligation to Mr. Morgan, of Guy's Hospital, for introducing this plan of treatment. We have repeatedly practised it with invariable success, and believe that it only requires to be more generally known to be almost universally adopted.

The annexed cut shows the curve of the sound and catheter we are in the habit of using. It differs from that described in Mr. Morgan's work, and if any one will take a skull and the two instruments, he will find that the one here described passes with much less catching against the walls of the passage. We also find this advantage hold good upon the living subject. Of course some little variation of the curve will be required according to the formation of the passage in different patients; but, as a general rule, we prefer the curve here described. The instruments are of silver.

1 Lateral view. 2 Shape of handle.

In an acute case, as a general rule, the opening on the face need never be made. Leeches, &c., are applied, and the passage freed by the probe and injection of warm water by the catheter. In a chronic case of fistula the same mode of keeping the duct open must be employed, either for the cure of the fistula, or, if the patient have worn a style, to enable him to dispense with it. The sound is first passed along the floor of the nose, its point directed outwards, until it lies fairly below the inferior turbinated bone. By turning the flat handle the point is then directed directly upwards, and moved gently backwards and forwards along the inferior surface of the turbinated bone, until a little cartilaginous ridge is felt. This is the edge of the orifice of the duct. The point is glided over it, and then, by depressing the handle, the instrument readily glides along the duct. No force must be used, as the bony structures around are very delicate. If much resistance be met with from an old stricture, the point only must be passed into the stricture, and allowed to remain a few minutes. By repeating this daily, the obstruction is gradually, if slowly, overcome. In one case we found it necessary to pass the instrument daily for upwards of a month. Assistance is also derived from cleaning the passage by the injection of warm water through a catheter of the same size and curvature as the sound. When once the passage is free, the tears descend, and all danger of a return of the disease is prevented by teaching the patients to pass the instrument for themselves. This they soon do with a little practice before a looking glass. The sound may be used once or twice a week, and the catheter daily. It is seldom safe to omit their use altogether, as collections of viscid mucus, or recontraction of the duct, are very liable to occur, and the short time occupied at the dressing table is a very slight set-off against the relief obtained by leaving off the style, or avoiding its insertion altogether. We do not hesitate to say that any surgeon who once practices the nasal method of dilatation, will for ever banish the style from his instrument case. But we must return to the work before us, the succeeding chapter of which is on the closure of openings in the air-passages.

TRACHEA. Small openings in the trachea are treated on the same principles as other fistulæ ; larger ones either by paring and loosening of the edges, and union by insect needles and twisted suture. Sometimes lateral incisions, three fourths of an inch from the edge of the opening, are necessary to assist the sliding of the integument. Interrupted sutures are better than the needles when the surrounding skin is very thin ; and in this case the lateral incisions must be two inches long. Transplantation of a flap is not to be recommended, as the wound is then larger and more complicated ; and the flap, after its separation, falls together between the fingers like a piece of wet paper, unites with difficulty around the thin wounded edges, probably dies, or contracts upon itself, and remains like a little ball at one side of the opening.

SCROTUM. *Oscheoplastice,* or restoration of the scrotum, is seldom required, but it is occasionally from the consequences of extensive burns, or of infiltration of urine. The cases in which Dieffenbach has operated, have been where one side of the scrotum was quite destroyed, and the testicle lying free and uncovered, or more or less covered by cicatrices. The skin of the scrotum is divided around the hard edges of the defect, avoiding the spermatic cord and the opposite tunica vaginalis; then the hard cicatrix is completely removed, the cellular tissue of the under border of the scrotum loosened, and it must be seen if this be sufficient for the complete covering of the testis; if so, the cicatrized surface of the testis is pared with the knife to the thickness of a sheet of paper, "as one would pare a lemon." Then after the bleeding has ceased, the edges of the skin are drawn over the testis, and the wounds united by a quantity of insect needles. Cold applications are used, then lead lotions; the needles remain until the fifth or sixth day, and then plaster is applied.

In two cases of total destruction of the scrotum, Bürger and Labat have restored it by flaps formed partly from the abdomen, partly from the inner side of the thigh. Dieffenbach does not seem to think the operation likely to be often successful, but says he would rather do it than remove a testicle.

PREPUCE. Seven pages follow on the formation of the prepuce, *posthioplastice,* which we do not think it necessary to analyse. We are more in the habit of removing than of making prepuces in this country. The latter operation can be scarcely ever *necessary,* and we imagine few Englishmen would desire its performance as a *Schönheitsoperation*—Anglice, for ornament. The Germans, however, apparently have peculiar notions on this point, as we have a chapter on the means of improving the form of the glans penis, to which we would refer those of our readers who are curious on the subject, and pass to the more important plastic operations upon the urinary passages.

URETHRA. We must refer to our Seventh Volume for the operations upon urethral fistulæ, where, at p. 399, will be found woodcuts and a description of the treatment of suture; and at pp. 413-14, of that by sliding or displacement, also with illustrations. Seven other operations are described in the present work as means of closing large defects of the urethra by transplantation of skin. In the first, the skin is taken from the scrotum; a large catheter is passed, and the edges of the opening so cut that a square opening remains, the points of which look towards each side of the penis; then a fold of the scrotum behind the opening is raised and cut through to the extent of two inches in length, and the wide bridge thus formed between the incision and the opening is loosened from its base. It is then drawn forwards, and its anterior border united with the wounded edge of the skin of the penis by twisted suture. A bougie is worn to prevent infiltration of urine.

The second operation is not to be recommended. The penis is kept near the left inguinal region, and a flap taken from thence and fixed as before.

The third is an annular transplantation of the prepuce, over openings in the anterior part of the urethra. This explains itself; the outer lamella of the prepuce is alone used.

Fourthly, we have annular transplantation of the skin of the penis, over openings in the urethra, close behind the glans. Dieffenbach has performed this three times with success, once having failed from subsequent erections.

Fifthly, the prepuce may be transplanted over openings of the urethra behind the glans. This is the best operation when the under surface of the glans has been destroyed by chancres, and the urethra opened, the contraction of the cicatrices having drawn the openings beneath the prepuce.

The two following operations we need not describe: one consists in dividing the prepuce at each side, and using the inferior segment to replace defects of the glans and urethra; the other, in bringing the skin of the back of the penis to its under surface, to close extensive openings in the urethra. One of the peculiarities of Dieffenbach's practice, however, must not be passed over. In order to insure the success of extensive urethroplastic operations, to prevent infiltration of urine, and procure union by first intention, he recommends opening the urethra in its posterior part, and drawing off the urine from this opening by means of an elastic catheter passed into the bladder. It is often seen that when two openings exist in the urethra, one before and the other behind, that the anterior one readily heals by cauterization or suture, provided the posterior one is sufficiently large to allow the escape of all the urine. This is still more certain when a catheter is passed into the bladder. When the posterior opening is large and behind the scrotum, the anterior part of the urethra remains naturally perfectly dry, and the opening then, even if it be an inch long, is readily healed. This points out the means of obviating the difficulty afforded in these operations by the urine; but the opening should never be made in case of small fistulæ, as thus we should heal a small fistula and probably produce a large one. It is only to be done in cases of great loss of substance, to ensure the success of the reparative operation, as the advantage is then on one side, the large opening being closed and a small one formed.

The artificial opening heals most readily when it is made by transverse incision. A large elastic catheter is passed; skin and cellular tissue divided; the under half of the urethra and somewhat more than the half of the catheter divided transversely; the catheter held with forceps, divided; the anterior portion withdrawn; the posterior drawn about an inch exterior to the wound; threads passed through its edges, and by them it is fixed to the soft parts with adhesive plaster. The orifice is stopped by a plug, and the urine allowed to escape every two or three hours.

When the cicatrization of the plastic operation is completed, the artificial fistula must be healed; and this is generally done without difficulty by keeping a catheter in the bladder, passed along the whole length of the urethra, and frequently cauterizing the opening.

The operations for the cure of hypospadias and epispadias, of course resemble those for the cure of urethral fistula, provided the canal be pervious; if not, a canal may be formed by transplantation.

"A fold of skin is seized at each side of the penis, and drawn together over the spot where the urethra is to be formed; then the borders of the fold are drawn together along the whole length by means of transfixion with a straight needle and waxed thread; an incision is then made at each side of the penis, through the skin, to prevent tension; and lastly, the borders of the folds which were tied together are cut away over the sutures, one after the other, with a pair of sharp scissors, and the wounded edges brought exactly together by means of a continued suture, or a quantity of insect needles, so that epidermis is in contact with epidermis. That the new canal may not be exposed to the urine, this is drawn from the old opening by a catheter. When the formation of the canal has succeeded, and it is also internally covered with epidermis, it must be opened forwards, and continued through the glans. If the glans have a cleft or fissure, this must be healed by the suture, and then the canal, which is still closed anteriorly, must be opened by a sound introduced from behind, and a bougie carried through the glans. The external opening between the glans and canal is closed by pencilling it with tincture of cantharides. Should the glans be imperforate, a small trocar must be passed through it into the urethra; then by means of a leaden sound the internal skinning over of the passage must be sought for.

"The closing the opening through which the urine has passed, by caustics and other means, concludes the treatment. This is impossible without introducing a catheter twice a day from the glans into the bladder." (pp. 540-41.)

VAGINA. This brings us to the chapter on *Vesico-vaginal fistula.* Did our space permit, we should gladly extract the graphic description our author gives of the miserable situation of a poor woman subject to this condition; but as we confine ourselves to his practical and novel observations, we pass over this, and his remarks upon its causes also.

These fistulæ vary greatly in form, size, and situation. Small ones can only be detected by the speculum. The quantity of urine which passes by the fistula varies with its size, and the power of retaining the urine in the bladder with its height. Should the opening not be detected by the speculum, the vagina is to be plugged by a roll of linen, and water, coloured by boiling black cloth in it, is to be injected into the bladder. A black spot on the linen plug will then point out the situation of the opening.

The difficulty in treating these cases arises from the feebleness of the plastic process. The wounded edges of the opening very soon become re-covered by a thin skin, and the vegetative process of agglutination, or even the production of granulations, is seldom established. This is the great cause of the non-success of simple cauterization; and it must be observed that unless the opening is *perfectly* closed, no good has been effected, as the urine dribbles through a small opening in sufficient quantity to keep up the miserable condition of the patient. Thus the numerous cases recorded where a large fistula is said to be cured, only a very small opening remaining, *which is expected soon to close,* are not cures, as the really difficult part of the cure remains to be accomplished.

If *cauterization* be frequently repeated, the parts surrounding the opening become often converted into a hard callous substance, and the subsequent healing by ligature is rendered more difficult. If milder caustics are used still oftener, no change is effected, as the reaction very soon subsides. Should cauterization be obstinately continued in the hopes of exciting granulation, loss of substance follows, and the opening becomes much larger than before.

Sometimes the *suture* is unsuccessful; the threads cut through one edge from the second to the fifth day, and all hang by the other; or some cut through one edge, some the other; the former is more usual. The small fissures made by the sutures do not unite. Sometimes the failure arises from excessive inflammation around the sutures, followed by mortification, and the opening is then also larger than before.

The stories of cures of these fistulæ by vaginal plugging and keeping a catheter in the bladder, are of doubtful authority. Dieffenbach has tried the plan, and found that the catheter does not prevent the plug becoming soaked with urine, and irritating the parts still more. A catheter cannot prevent the passage of urine through the fistula, and this is the great impediment to union of the pared edges by the first intention. The edges may be perfectly approximated and lymph effused, but its organization is stopped, and the edges are soon seen to be covered by a fine ash-coloured coat of mortified cellular tissue. A single drop of urine between the edges is enough to prevent their union.

A natural cure is sometimes attempted by prolapse or protrusion of the mucous membrane of the bladder. In one case, where this had formed a plug to the opening, but was only partially adherent, Dieffenbach completed the adhesion by the application of tincture of cantharides to the ununited portion.

Whatever operation may be decided upon, the surgeon must first make himself thoroughly acquainted with the situation, &c., of the opening in every position of the patient, as well as if he could have it always before his eyes. A castor-oil laxative is given the day before the operation, and shortly before it an enema of warm water, and the vagina and bladder are to be thoroughly washed by repeated injections of large quantities of warm water. The most convenient position for the patient (and the surgeon) is lying upon the back, and not upon the abdomen, as has been recommended. She lies near the edge of a table, which is placed close to a window; the upper part of the body only moderately elevated; the buttocks project over the edge of a hard horsehair mattress, so far that the patient would slip off if she were not held. The operator sits on a stool between the widely-opened thighs; the knees are bent, and the whole limbs raised and bent back as far as possible by an assistant on each side. Pieces of sponge of the size of a walnut are prepared before hand, and by means of straight forceps the urine is thus repeatedly absorbed. After a long account of all the instruments used by different surgeons in these operations, Dieffenbach quaintly remarks that they "only require clockwork to operate by themselves." The various modes of operating which have been recommended are very clearly detailed, and this chapter may be considered as a complete treatise on the subject; but as our present object is to give an account of the author's practice only, we shall do so as shortly as possible, without following his remarks upon the practice of others.

1. *Operation by common suture.* The fistula of moderate size in the anterior part of the vagina; patient prepared and placed as described; three blunt hooks are introduced, and used one on each side to separate the labiæ, the third to draw the commissure backwards; a sharp double hook is then fixed in the anterior part of the fundus of the vagina, the rugæ drawn level, and the part of the vagina in which the opening exists is drawn into sight. The external border of the opening is probably smooth and hard, and the internal covered by a fine red edging of the mucous membrane of the bladder.

"While an assistant holds the hook which draws the vagina into view, the edge of the opening is seized by a conjunctiva hook; it is transfixed with the point of a fine scalpel half a line broad, and cut all round, so that a little more is taken away from the external than from the internal edge, and the wound has a wider external than internal opening, and is somewhat pointed from before backwards.

The internal circular line runs round the fixed point of the mucous membrane, none of which is to be cut away. The part of the ring now first hooked is loosened in a quarter circle; then the hook is again fixed in the neighbourhood, incision carried on further, and this is repeated all round, and the edge resembles a fine circle of skin. The fixing always answers better with the hook than with forceps; it is only when a strip of the ring is to be removed that this is fixed with forceps, and the point of the knife carried round the opening. Then cold water is injected into the vagina, and this is dried again with pieces of sponge, introduced by polypus forceps." (p. 557.)

Three or four sutures are necessary; curved needles are used. The left edge of the opening is first transfixed at the distance of two or three lines, the needle carried close to the mucous membrane of the bladder, and drawn by forceps so far that the middle of the thread arrives at the opening; then the needle is carried across the opening, the other lip of which is pierced close to the vesical mucous membrane, and the needle brought out so that the two threads hang at the same length out of the orifice of the vagina. The wound is again cleaned and dried, and a brush carried over it wet with diluted tincture of cantharides, to obviate the influence of the urine, and the threads are then tied together tolerably tight with a double knot. The ends of the suture are taken in the left hand, and the tied part drawn lightly forwards, so as to project a little, and then the approximated lips of the wound are both transfixed at the same time, the thread passed and tied as before. This is the middle suture; the posterior is made in the same manner; the threads are then cut short off close to the knots, leaving one end of the anterior suture only, which must be cut off close to the labia. The vagina is again syringed with cold water and dried with sponge, and then plugged with soft charpie, which is to be saturated with wine by placing the point of a syringe in the charpie. A large male catheter is passed into the urethra, the patient laid upon her back, and the open catheter connected with a receptacle placed between the thighs in order to keep the bladder empty. Several times daily the bladder must be syringed out with cold water, which is immediately allowed to run off.

Three days afterwards the charpie is to be withdrawn, and the vagina syringed out with warm water, and the sutures examined by careful separation of the labia. Should they not be visible on account of swelling of the parts, and still the vagina is free from urine and has no unpleasant odour, nothing more is to be done than introduce soft dry charpie into the vagina. The following day the sutures must be brought into view by placing the patient on an operation table and separating the labia, and then the short ends of the two posterior sutures are seized with forceps, divided, and removed; the anterior knot is left till the following day, and then removed; charpie is introduced into the vagina, and then saturated by syringing with infusion of camomile. The urine passes some days longer by the catheter, and about the eighth day its voluntary emission may commence; then the patient goes about and is cured.

"This is the progress of a successful case; but when it does not go on well, and one or more sutures have cut through, which generally happens about the third or fourth day, the nose tells before the eyes what has happened; instead of wine, the urine is smelt, and when the charpie is withdrawn, the urine is detected by its intolerable odour. The vagina is to be cleaned by injections of warm infusion of camomile, the seat of operation examined, and the sutures which lie loose, or have cut through one edge, are to be removed. Sometimes all three sutures have cut through, and must be removed, and the opening appears as large as at the time of operation. If partial union of the opening has taken place by means of one or two sutures, these must be left a couple of days, and the edges irritated, as will be explained when speaking of cauterization. No charpie must be introduced, as it would become moist with urine, but the vagina is to be frequently syringed with the camomile tea, and the urine passes off by the catheter. If the remaining opening does not close by cauterization, the operation may be repeated after the complete recovery of the patient from the first." (pp. 558-9.)

In cases where the fistula is situated very far forwards in the vagina, the sutures are better passed from before backwards, than from right to left. In all other particulars the operation is the same as that just described.

When the fistula is deeply situated, for instance, in the middle of the fundus of the vagina, the difficulty of the operation is of course greatly increased. It is however possible; either—1, by strong traction forwards of the upper part of the vagina; 2, by operating by the guide of the sense of touch; or 3, by the speculum. The first is preferable, if practicable, as the presence of the speculum considerably increases the difficulty of paring the edges and passing the sutures. The operation unassisted by the eyes is very difficult: "it requires much practice and great imagination; one must think himself blind, and have eyes on the points of his fingers." It is not to be attempted if either of the other modes can be employed. The general plan of the operation is that just described; slight modifications are of course necessary, but these are so evident that we need not describe them.

*Union of fistula by running suture.* This is to be recommended when the fistula is small, the edges soft and yielding, and particularly when it is situated in the anterior and middle part of the fundus vaginæ.

The patient is placed and vagina kept open as before, and its upper wall brought far forwards by a double hook, and the edges of the opening pared to the thickness of a sheet of paper; and the day before inflammation must have been set up in them by concentrated tincture of cantharides. A small strong curved needle armed with waxed silk is passed two or three lines from the edge all round the opening, passing it inwards and outwards, and in and out again, until the opening is stitched all round at the same distance from the edge, and the needle with the thread comes out again at the point where it was first inserted. The thread must lie between the mucous membrane of the vagina and that of the bladder. The edges are then smeared over with tincture of cantharides, and the thread tied. One end is cut short off, and the other brought out of the vagina. After the operation the vagina is plugged with dry charpie.

The advantage of this suture is that it allows neither the entrance nor passage of urine so long as it has not cut through the parts, because the edges of the opening are completely bound together, and even when the operation entirely fails the opening is always diminished in size. It is also very safe, and may be frequently repeated, and the hope of success increases with every repetition, which is not the case with other methods, as after their failure the case is rendered worse than before.

*Union by twisted suture.* It is much to be regretted that this is only applicable to fistulæ situated in the anterior part of the vagina. When they are further back the common suture is preferable, as the edges are more readily brought together, and if success does not follow, they are less injured than by the needles. But in large fistulæ of the urethra or bladder in the anterior part of the vagina it is the most successful. The spot must be seen, as the needles cannot be applied by the feeling, or through a speculum.

The patient is placed as usual, and the upper vaginal wall drawn as far forwards as possible with a sharp hook. The edges of the opening are fixed by the hook and pared with a scalpel. A strong insect needle, from which a part of the blunt end has been snipped off, is fixed in the needle-holder at right angles, and by this carried through both the wounded edges of the anterior angle of the wound. The points of puncture and exit of the needle are four or five lines from the edges. Then a thick pitched (gepichten) double thread is twisted round the needle once, the needle is a little curved, then the twisting is repeated a few times, and both ends of the needle are snipped off a few lines from the thread. Then the ends of the thread are given over to an assistant, who therewith draws the fistula a little forward, in order to further approximate the edges of the wound, and facilitate the application of the second needle, which must be placed three lines from the first. This is then surrounded by thread like the first, and as many more needles are then passed as are necessary for the closure of the opening. The ends of the most anterior needle only are left a little projecting, the others are all cut off close to the knots. At the close of the operation a double pitched thread is twisted around the collection of sutures, so as to form a sort of ligature similar to that of a small prolapsus around the part occupied by the suture, and thus retain the urine from the inner surface of the wound. The vagina is then cleared and plugged with charpie, which is left unmoistened in young persons; in strong powerful persons it must not be filled, but several times daily cold water is to be injected into the bladder, and the catheter left there constantly.

The examination of the parts must be carefully undertaken after some days, and the needles withdrawn by forceps. If one needle has completely cut through, it is removed and the others left; but if it have only become loose, a new thread must be twisted round it. If all the needles have become loose, and the urine again passes through the opening, they must all be removed, as no union can be expected from this operation; and by the needles lying longer, and being again surrounded by the threads, the borders of the opening would be destroyed. If only a small opening remains, it must be treated on the principles before described. If the operation entirely fails, the hope of better results at a subsequent period must support us.

*Union after cauterization.* We need not describe this operation. The hot iron is preferable to any other caustic except the cases to which the tincture of cantharides is applicable. In general terms, for the reasons before stated, Dieffenbach prefers the other modes of operating, but says that the remedy is "a great one, and that in the most difficult cases." In two cases of fistula, close to the neck of the uterus, through one of which fistulæ the little finger, and through the other a thick catheter could be passed into the bladder, union followed one cauterization. "I scarcely trusted my nose, my eyes, or my fingers, when I found the opening perfectly closed. In a *tolerable number* of cases, perfect healing followed the long-continued application of cantharides ointment, or a repetition of the cauterization." (p. 572.)

Transplantation has also been practised: 1, from the wall of the vagina; 2, from the neck of the uterus; 3, from the bladder; 4, from the labia; and 5, from the skin over the glutei.

Transplantation of part of the vaginal wall can scarcely ever give much prospect of success, and is principally employed to diminish openings which are afterwards cured by other methods. The neighbouring part of the vagina is loosened and drawn over the opening like a bridge, and then a row of sutures of lead wire are passed with palate needles, drawing the needles from behind forwards with forceps. Then the sutures are drawn together until some tension commences. Then incisions at each side are made through the vaginal parietes somewhat larger than the opening, and the sutures are again drawn more closely together. When tension is again produced, the side flaps are loosened as far as necessary to allow of union. Then the sutures are cut off a quarter of an inch from the ring. The after-treatment is the same as after other operations. Probably the large opening is converted into a fissure, and this is to be treated by the suture or cauterization.

Dieffenbach has never followed the example of Horner and Le Roy d'Etiolles by transplanting portions of the neck of the uterus.

Portions of the bladder can only be used when they prolapse through the opening. In this case, as before stated, their union may be completed by cantharides.

Transplantation from the labia was practised by Jobert: his paper will be found in our Second Volume, p. 561.

Dieffenbach has never transplanted the externa integument. Recorded cases have been generally unsuccessful.

*Complications.* Vesico-vaginal fistula may be complicated by—1, rupture of the neck of the uterus; 2, of the perineum; and 3, of the rectum. In some cases all these evils are combined.

1. When a part of the neck of the uterus is torn, and is connected with one border of the fistula, the union of this part must be attempted by the suture or cauterization. If its whole length is split, and the fissure is continued into the bladder, or into the vagina also, and the urine escapes by the whole opening, the suture is less useful than the cautery. In applying the iron the os uteri must not be cauterized, or its adhesion and closure would follow. The suture is not to be recommended, as its application is very difficult in this situation, and very free granulation follows cauterization of the cervix uteri.

2. When the perineum is ruptured, a vesico-vaginal fistula is generally very large, as a very severe labour has probably been their cause. Prolapsus of a portion of the bladder is then frequent, and the uterus sinks and afterwards prolapses. Operation upon the fistula is only to be attempted when it is small enough to afford reasonable hope of success, but if it is so large that prolapse of the bladder has taken place, then the surgeon must content himself by healing the perineal rupture. The sunken uterus and bladder must be supported during the healing of the fissure after the application of the suture, and cold water frequently injected to prevent the irritating effects of the urine. If both operations are to be performed, that upon the vesical opening must be first performed, and when that has succeeded the perineum must be attended to.

3. When the perineum and rectum are both ruptured, the case is most melancholy. The contemporaneous rupture of the bladder, vagina, perineum, and the inferior part of the rectum forms a large cloaca where urine and excrement mix. Both bladder and uterus are sometimes filled with excrement. The treatment is exactly the same as when the perineum only is ruptured.

*Closure of the os vaginæ* has been recommended by Vidal de Cassis in cases of large incurable vesico-vaginal fistulæ. The anterior part of the vagina is made to adhere, and the canal changed into a urinary reservoir. M. Vidal has been much blamed for his proposal. Dieffenbach thinks without reason, as the condition of the patient is ameliorated, and especially if the perineum and rectum be also ruptured, the uterus prolapsed, and we are thus enabled to heal the whole enormous fissure.

"The closure of the vagina is only applicable to the largest perforations, when no healing can be expected. It has the advantage of supporting the bladder, and preventing the continual escape of urine. The fear that the urine retained in the vagina will cause inflammation there is altogether groundless. It is seen in every case of vesico-vaginal fistula that the vagina, moistened with urine, appears pale, and never irritated, while the bladder seems red and inflamed by the air. It is only upon the outer surface of the labia, particularly upon the skin over the glutæi and that of the inner side of the thighs, that the urine irritates and keeps up a painful chronic inflammation." (p. 597.)

The fears of the passage of the urine through the uterus and tubes into the peritoneal cavity, and the formation of a vaginal calculus are equally futile. The operation is not to be regarded as a means of *cure*, but as an artistical amelioration of the unfortunate condition of the patient.

The operation is exactly that of ruptured perineum, except that lateral incisions of the labia must be carried up to the height of the clitoris. When the application of the needles and twisted suture is accomplished, a catheter is passed by the urethra, or by the small opening left free, and cold water copiously injected, and again allowed to run off. The catheter remains for the passage of the urine, and the application of cold lotions. The sutures are gradually removed, commencing with those which have cut through one edge. If the suppurating edges completely separate from each other, an irritating ointment is applied and, when they are cicatrized, granulation is to be sought by cauterizing. The running suture may then be used to diminish the opening. If partial union follows the first operation, the portions remaining open are to be cauterized or irritated by tincture of cantharides or red precipitate, until union is perfect.

In one case, in which Dieffenbach thus closed the vagina, great advantage was obtained. A small opening for the urine was left, which the patient could close by a small plug, and thus retain the urine. On removing the plug she had the power of voiding a large portion of her urine. The formerly irritated and fiery red nates and thighs were covered with healthy skin, and no trace of any injurious influence of the urine upon the bladder or uterus was observed. But this is the only successful case on record. Vidal himself did not complete the cure. Velpeau, Lenoir, and others, have also failed.

Dieffenbach is of opinion that no mechanical recipient for the urine is of any use. Still, patients wear useless and often injurious contrivances for years, satisfying themselves because *something is done.*

*Recto-vaginal fistula* is closed with greater ease than the vesico-vaginal opening, but still much difficulty arises; 1, from the thinness of the ruptured parts; 2, from the double coating of mucous membrane; 3, from the passage of excrement and flatus from the rectum. The causes are difficult labour, foreign bodies in the rectum or vagina, abscesses, &c.

The modes of operation are, 1, suture after removal of the edges; 2, suture after cauterization of the edges; 3, cauterization alone.

If the fistula be a species of large fissure the interrupted suture is the best. It is necessary to have small strong curved needles, silk thread, a fine small hook, a thin pair of hooked forceps, and a needle-holder. The rectum is cleared by enema, and then well washed by injection of cold water, and the patient placed in the same position as described for the operation upon vesical openings. An assistant with his finger, or a sort of leather bougie in the rectum, presses the gut and vaginal wall downwards and forwards. The edge of the opening is then fixed by the hook, and pared with the point of the scalpel, separating the firm connexion between the rectum and vagina. The needle is held at right angles in the needle-holder, the inferior pointed part of the opening drawn forward with the hook, the needle carried through both edges, and the suture tied tolerably tight. The ends of the thread are drawn a little down, and then the second suture is passed and tied, and so on until the highest one is tied. Then all the ends of the threads are cut off near the knots, except the lowest, which is only shortened, and allowed to hang between the labia. The inferior portions of the rectum and vagina are to be stuffed with soft charpie, particularly the former, to keep away the excrement from the sutures.

After the operation but little food is to be given, and constipation is to be obtained by doses of opium. If the charpie in the vagina is moist it is changed the following day, while that in the rectum is allowed to remain. The sutures are removed, one one day, another the following, and so on; and the charpie kept as long as possible in the rectum. When necessary it is removed with forceps, and then warm camomile tea is injected through a thick elastic catheter, and allowed to pass off again. If the operation has partially or entirely failed, the sutures are removed, the edges cauterized to some extent with the nitrate of silver, and the vagina and rectum plugged with charpie, which is changed daily. Small openings are thus closed; if larger, a repetition of the sutures becomes necessary.

In small fistulæ the running suture after cauterization of the edges is the best practice.

"The nitrate of silver is passed through a small speculum in the rectum, and the border of the fistula and the surrounding parts for a quarter of an inch in extent are touched with it. On the vaginal side inflammation is excited by a ball of charpie moistened with tincture of cantharides, which is prevented from touching the other parts of the vaginal walls by plugging with dry charpie. If on the following day the edge and its circumference to the extent of half an inch is properly inflamed, the epidermis is raised by forceps and rubbed off with dry charpie, and then the running suture is applied by means of a fine curved needle and a needle-holder in the usual manner, carrying the needle all round the opening between the surface of the rectum and vagina, and tying the threads moderately tight together. The rectum and vagina are plugged with dry charpie. If union is accomplished the suture is divided and removed on the fifth or sixth day; if the operation has this time failed, the opening after cicatrization will appear much smaller than before, and by one or more repetitions of the operation will probably be closed." (p. 605.)

By one or other of these methods, Dieffenbach has cured many of these fistulæ. Some were an inch in length. Others remained after union of ruptured perineum when the rectum had been torn at the same time. In one case the opening, although not completely closed, was so small that no particle of excrement, only flatus, could pass.

The result of simple cauterization is very doubtful.

PERINEUM. We now pass to one of Dieffenbach's particular studies, Union of ruptured perineum. We may refer to an abstract of some of his cases in a former number, (Vol. VI, pp. 536-7) and to some remarks on the same subject, (Vol. XI, p. 195) and now proceed to detail his practice a little more fully.

In old cases of perineal rupture union is effected by suture after paring the edges; in recent cases the paring is not necessary. The only contra-indications to the operation are approaching menstruation, pregnancy, profuse leucorrhœa, diarrhœa, disease of the general system, and severe local inflammation.

Dieffenbach was formerly of opinion that it was better to wait some time after delivery before attempting this union, as the parts were generally much bruised and torn, the patient's system depressed, &c.; but experience has now taught him that it cannot be done too soon; that the first twenty-four hours after delivery must be chosen, and this especially when the rectum is also torn. When the suture is immediately applied union occurs in the majority of simple cases, and if the rectum be also torn, a recto-vaginal fistula only remains, which is treated on general principles.

The necessary instruments are thick strong needles, bent to a three-quarter circle, and threaded with waxed silk doubled twice; insect needles of various thickness and length, and cotton threads; a strong needle clipper; strong forceps hooked and smooth; pincers, knives, scissors, water, sponge, charpie, and syringes; the patient prepared as for vesico-vaginal operations.

*Partial recent rupture.* Suppose the anterior half of the perineum be torn through, and the wounded edges are even, the left edge is held with the thumb and finger of the left hand, and the curved threaded needle is inserted a few lines from the posterior angle of the wound, and about a third of an inch from the edge; it must pass straight, and not obliquely, through the wounded edge close to the vaginal mucous membrane, and be drawn forward with forceps until the middle of the thread arrives in the wound; then the right edge is seized, everted a little, and transfixed at the point exactly opposite the passage of the needle through the left edge; the threads are then tied tolerably tight with a double knot, and the ends cut off; then a second suture is applied, and lastly the most anterior one, so that the commissure may be exactly united, and the edges of the mucous membrane be in exact apposition. Three sutures are generally sufficient for a perineum torn half through; but if after their application the space between them gapes, a couple of fine sutures may be applied, which merely go through the skin.

*Total rupture.* When the perineum is torn completely through to the anus, the edges must be made even, if torn muscle or cellular tissue render it necessary, and then the suture passed as before; but the most posterior suture must be formed of four or six strong waxed silk threads, passed a full half inch from the edge, and through the whole thickness of the wound, the vaginal mucous membrane excepted. Five other sutures must be applied, the middle being the strongest, and their distance from the edge of the wound gradually diminishing to one third and one fourth of an inch; union is thus more exact. The edges of the mucous membrane at the commissure are united by five sutures, and a couple are also applied on the internal surface.

*Partial complicated rupture.* The posterior or middle part may be torn, the commissure remaining; to these the preceding directions apply. The rupture may not be in the median line, but at one side, and complicated by injury of the labia, ecchymosis, &c.: in such cases the edges must be carefully made even by scissors before applying the sutures.

In total rupture, complicated by rupture of the anus and inferior part of the rectum, union must be attempted as soon as possible, or the large wound will become inflamed by the urine, lochia, and excrement; the cellular tissue sloughs, and unhealthy suppuration comes on; and if granulation and cicatrization succeed, much substance is lost, and the operation is rendered more difficult and painful. In such a case union of the rectum is first attempted by fine needles, and fine single waxed silk ligature, forming the *un*interrupted suture. The ends of the thread are brought out by the anus, then the perineum is united as before.

*Old total rupture.* The operation here differs, inasmuch as the cicatrized edges have to be removed before the sutures are applied. If much difficulty occurs, from contraction of the parts, in approximating the edges a lateral incision may be made on each side.

If there be also rupture of the rectum, the excrement passes through the large fissure, and, on examination, it appears as if there were no perineum whatever, for its halves are drawn to the surrounding skin, and very often scarcely any cicatrix is detected in their room. Sometimes, instead of a perineal fissure, some cicatrized folds remain on each side, as the remnants of the perineum. Here the operation will vary according to circumstances: either 1, union of the fissure; 2, the same with lateral incisions; 3, transplantation of neighbouring skin. We need not recur to the first and second method. The formation of a new perineum is only to be undertaken in large old fissures, reaching deep into the rectum, where the external skin is cicatrized and deeply wrinkled. The edges of rectum, anus, and vagina are pared. "Then in the depth of the furrow two parallel incisions are made of the required breadth of the perineum; an oblique incision an inch long is carried from their termination outwards, one both before and behind to each incision. Thus two oblong quadrangular flaps are formed, which reach from the labia to the anus, and which are then loosened from their under surface." (p. 626.)

This sort of bridge is united by sutures, and if there is tension, this must be relieved by lateral incisions.

The after-treatment is of the greatest possible importance with regard to the success of these operations. The best position is upon the back, thighs moderately open, and knees bound together with a thick compress between them. If they are bound together without the compress, the united parts are pressed too much together, and this, in wounds united by suture, is almost more injurious than the opposite fault. For the same reason the position on the side is not to be recommended. In fresh ruptures the parts are often washed by warm-water injections, and dried with sponge. If there is much collection of moisture in the vagina, charpie must be introduced and often changed. If much inflammation follow the sutures, leeches must be applied, and antiphlogistic general treatment adopted. In old cases, if the woman be strong, cold applications are used, and often changed; if she be old or weak, lead lotions. The urine is never to be passed voluntarily, but always drawn off by the catheter, until the wound is firmly cicatrized. After three days a ligature may be removed, a couple on the fourth, the middle and posterior being left the longest. The internal sutures of the mucous membrane are withdrawn, when it can be done without stretching the parts; the uninterrupted suture of the rectum must be left to come away of itself, and sometimes requires a fortnight. Generally speaking, after the third day, lead lotions are useful. Everything must be done to prevent the patient having a motion for six or eight days after the operation: spare diet, and a quarter of a grain of opium twice daily. When the union is firm, the rectum is cleared by injecting warm barley water through an elastic tube, assisting the passage of scybala by a scoop; better diet is then given.

If union follow around some of the sutures, and a fissure remain in other spots, charpie, wetted with infusion of camomile, is placed in the fissure, and often changed. If the edges are indolent, nitrate of silver and dry lint are used. If a recto-vaginal fistula remain, it must be treated as before directed. If union does not follow the application of nitrate of silver or resinous dressing, a second operation must be performed after cicatrization and restoration of the general health. After the most successful operations, the vagina must long be kept well cleansed with warm water, and a mild ointment applied to soften the cicatrix. Very often the hardness entirely disappears, and after some years, scarcely any trace of the operation remains. T bandages, sponge, or pessaries in the vagina, daily evacuation of the bowels, which have all been recommended, are excessively dangerous and injurious.

The manner in which this subject is treated is most creditable to the author, and the results of his practice are most encouraging. He says, "Several women upon whom I have operated had suffered the rupture in the first labour, ten or fifteen years before, and had since remained childless; after the union of the perineum they conceived and bore children, and in no case did a new rupture again take place from the labour." (p. 637.)

Is not this a triumph of modern surgery? Is not this sufficient to reconcile the most sceptical to endure all the uncertainties and failures of the art?

UTERUS. The operations for the cure of *prolapsus of the uterus and vagina* are contained in the 57th chapter. Palliative treatment by pessaries, or sponges impregnated with astringent fluid, is of use when the patients are very weak and there is great relaxation of the parts, but in the majority of cases mechanical support does much more harm than good, and the radical cure must be attempted, unless the party is advanced in life, very weak, or suffering from incurable disease of the uterus or vagina.

Sometimes a natural process of radical cure is set up by inflammation, ulceration, and subsequent granulation of the vagina, with thickening of the submucous cellular tissue, and contraction of the cicatrices. Prolapse of the uterus is thus prevented, and one of the methods of radical cure is an imitation of this process.

There are two principal methods of obtaining a radical cure. The first, *elytrorrhaphie,* consists in producing an artificial stricture of the vagina, by excision of parts of the mucous membrane of the projecting parts, or of that of the vagina. An elliptical portion three inches long and two broad, the points of which are above and behind, and below and before, is removed from each side of the vagina, and if the parts are very lax, a small segment may be also removed from the anterior wall; but generally it is better not to interfere with the anterior wall, on account of the danger of injuring the bladder, and it is better to employ subsequent cauterization if necessary. After the removal of the portions of mucous membrane, the edges of the wounds are brought together by sutures, and the uterus replaced; the parts are cleansed and the vagina plugged with charpie. The sutures are removed when the speculum shows that union has taken place; but even if union by the first intention does not follow, and the wounds suppurate, nothing is lost, as the prolapsus is often better supported after healing by granulation and cicatrization, than if the wounds had at once united.

A second method consists in paring long strips of mucous membrane from the vagina along its whole length, with the express purpose of producing strong superficial cicatrices.

After these operations the patient is kept recumbent, that the falling uterus may not interfere with the wounded parts; the catheter is at first kept in the bladder, and mild enemata are administered. Cold applications are injurious, either bringing on erysipelatous inflammation, or retarding and rendering torpid the granulating process. Injections of warm camomile tea are useful, and after some time charpie or sponge, moistened with red wine, may be kept in the vagina; and lastly, alum and other astringent lotions are used.

Dieffenbach, however, prefers cauterization of the vagina to either of these proceedings, as he says relapses are apt to follow them. When the parts are so large that the uterus immediately falls on the patient's standing erect, six lines of cauterization with a conical iron, passed slowly from the internal surface of the labia to the neck of the uterus, are required; if the parts are less lax, three or four lines are sufficient; then the parts are covered with cotton wadding, and the separation of the sloughs waited for; then a mild ointment spread upon charpie is applied, and when the swelling goes down the uterus is replaced.

When the uterus prolapses only after long standing or walking, or on going to stool, it is better to apply the iron by means of a speculum, than to draw the uterus down, as in the method just described. Cotton wool is introduced, after some days it is removed, warm milk or barley water injected, and cicatrization assisted by subsequent injections of lead lotion.

The results of cauterization are most satisfactory. Our author has thus cured a great number of cases, and enabled the sufferers to dispense with their pessaries. In some cases the parts were so much enlarged, that even the largest species of mechanical apparatus could not be supported, but fell out. Some of these persons after the cure conceived, and their labours were unattended by difficulty. In one case, after labour, the uterus again sunk, and a second slight cauterization was required. Cauterization is also much more safe than any of the cutting operations, and the iron is far preferable to the nitrate of silver, potassa fusa, or chloride of zinc, as these are seldom successful—either not acting to sufficient depth, or destroying the parts to an extent which is not desired.

This radical cure is complete. Fricke has employed a method of half cure, by causing adhesion of the labia, by paring their edges, and uniting by suture, *episiorrhaphie.* The plan is easy and safe, and prevents prolapse externally. It is thus a palliative rather than a radical cure, and has the disadvantage of rendering the patient unhappy; and if conception take place, redivision has been necessary to allow of the birth of the child.

*Prolapsus of the vagina* is the condition in which flaps or folds hang from the genitals externally: the uterus may or may not be sunk. The methods of operation are the same as those for prolapsed uterus, but here excision is more useful than cauterization.

The *gynoplastic* operation is treated in the next chapter,—the operation for opening or dilating the closed or contracted genital organs of the female. This is required in cases of total or partial adhesion of the labia or nymphæ, imperforation or fleshy condition of the hymen, membranous closure of the vagina high up, partial or total adhesion of the vaginal walls to each other, and in closure of the os uteri.

Adhesion of the labia and nymphæ in the first months of life are separated by mere pressure with the fingers, forcing them apart, and then keeping a piece of card between them. In adult girls, unless the union is so great as to impede the escape of urine and menstrual fluid, it is better not to interfere before marriage; but if the union be complete, or so great as to cause more or less retention of these fluids, division with the scalpel must be effected. The more superficial the union, the less must be the extent of incision.

When the adhesion is very extensive, simple division is sure to be followed by recontraction, and transplantation of skin or mucous membrane alone affords means of radical cure. Separation is effected by the scalpel, the mucous membrane of the posterior border of the wounded surface loosened, and then drawn forward and united by suture with the anterior border; or the external skin may be used. Incisions of this form are made from the posterior commissure towards the perineum ┌┴┐. The flap thus formed is loosened from its base, and separation of the labia having been previously effected, it is drawn inwards, and united by sutures with the wounded border of the mucous membrane.

We need not describe the mode of perforating or excising the hymen.

Membranous closure of the vagina, if any opening exist in the membrane, is treated by the probe-pointed bistoury; if the borders are very callous, five or six notches may be made. The parts are plugged with charpie, and sometimes it is necessary to wear elastic bougies for some time.

The operations, when there is total adhesion of the vaginal walls, are the same as those just noticed, but of course more difficult. The greatest care must be taken in the direction of the incisions, as Dieffenbach has seen incurable vesical and rectal fistulæ follow unskilful practice.

When closure of the os uteri is membranous, and the membrane is thrust forward by the menstrual fluid, the operation is without difficulty—division with a pair of scissors. If the lips of the os uteri are adherent, and the canal of the cervix also, perforation with the pharyngotome is followed by dilatation of the puncture with a probe-pointed bistoury. When the os uteri is closed by cancer, or in any way so that its opening is impracticable, and the patient is pregnant, if rupture of the uterus is feared, hysterotomy must be performed through the vaginal portion.

RECTUM. We have now a chapter on *closure of the rectum* and anus. This condition varies; the closure may be simply cutaneous, and simple perforation then suffices to afford a passage for the excrement. The rectum may not be sufficiently long to reach towards the integument; in this case a new canal must be formed. It may be too long projecting beyond its normal situation; in this case its opening must be obtained at the proper site, and the superfluous portion removed. Lastly, it may open in another cavity, vagina, urethra, or bladder; here the unnatural opening must be closed and another formed.

The formation of a new canal, when the rectum is too short, is effected, after catheterism of the bladder, by an incision in the situation of the anus half an inch deep, and then carrying on a trocar in the direction of the rectum to the extent of two inches, if necessary. If the gut is opened, it is cleared by injections of warm water through an elastic tube, and the opening kept constantly patent. If the rectum can be brought down to the external skin, it is to be stitched there.

When the rectum opens into the vagina, two methods of operation are laid down—the former easy, the latter more difficult, but its result more speedy. The former consists in passing a bent sound through the vaginal opening for a little distance up the rectum, inserting the knife close behind the opening outside the vagina, and carrying it through the skin close to the coccyx, without enlarging the opening into the rectum; then the rectum is laid bare, and its edges united with those of the skin. The vaginal opening is afterwards treated by caustic. After complete healing, the inferior anterior open perineal end is separated by transverse incision from the vaginal wall; the rectum thus drawn back into the cicatrized opening, and the new wound of the perineum united.

In the second method an oval portion of skin is excised in the natural situation of the anus, and the rectum freed both on its anterior and lateral surfaces; then a transverse incision, half an inch long, is carried through the anterior part of the perineum, close behind the commissure, up to the posterior wall of the vagina; the part where the opening of the rectum unites with the vagina is divided by fine scissors, and the separation of the perineal end completed by the scissors. The border of the freed rectum is then united by fine sutures with the external skin.

In all the cases of rectal opening into the bladder which Dieffenbach has seen, death followed from inflammation set up by the entrance of meconium.

*Stricture of the rectum* is treated in the following chapter. It may be superficial from ulcers around anus, and the contraction or adhesion consequent upon their cicatrization. Dilatation, incision, and sometimes excision of cicatrices, are necessary.

In some cases from inflammation, or cicatrization of ulcers of the mucous membrane, the rectum is drawn together as though it were surrounded by a ligature, and an opening, only sufficient for a quill to pass, remains in the centre. This is treated by passing a small knife along the finger and notching the cicatrix, and afterwards passing an oiled wax bougie. If the contraction return, and the stricture is not more than two inches from the anus, it must be extirpated. A curved blunt-pointed bistoury is introduced along the left index finger, and the anus enlarged to the extent of an inch and a half, above, and also towards the coccyx; the edges are then held apart by blunt hooks, and a sharp hook passed through the stricture, the ring of which is drawn down, and its base surrounded by incision with a small scalpel; the part included in this incision is then fixed by hooks and cut away with scissors. Between the superior and inferior broad portions of the rectum a chasm now remains, the superior and inferior edges of which must be united by seven or eight sutures, so that a fissure remains. The threads are cut away close to the knots, charpie introduced in the rectum, and the external wounds united by sutures; cold applications are employed, and the patient constipated by opium. The sutures of the rectum are left to separate of themselves. "In this manner I have completely cured many patients who had long been unsuccessfully treated by bougies, and in whom I had often incised the strictures in vain, and a relapse has never taken place." (p. 687.)

*Spasmodic stricture* has its seat in the external or internal sphincter. Narcotics and enemata are only palliatives; bougies make matters worse; and division of the sphincter is the only cure. This may be done in the common way, carrying the knife along the finger introduced into the rectum, or by subcutaneous incision, but the latter method possesses no advantage, as the wound in the other case always heals readily.

*Colotomia,* or the formation of an artificial anus, is the subject of the next chapter. Our author follows Amussat, and therefore we need not condense his descriptions at present, but take the opportuity of referring our readers to an excellent article on Intestinal Fistula, by Mr. Teale of Leeds, in one of the last published numbers of the 'Cyclopædia of Practical Surgery,' where this subject is most fully and satisfactorily treated, and pass on to the modes of curing preternatural anus by operation, which are described in the 62d chapter. This subject, however, is one of such importance that we think it better to devote a separate article to its consideration hereafter, than to pass over it here in a cursory manner.

FINGERS AND TOES. The 64th chapter includes the operations for the separation of fingers or toes, which have been united together either congenitally or in consequence of disease. In young children, when the uniting membrane does not quite reach the tips of the fingers, round smooth cords passed between the fingers like violin strings, and fastened to a bandage round the arm, by long-continued pressure sometimes effect a cure. Simple division with the scalpel is sufficient when the union is membranous and superficial, fine linen being wound round the finger after bleeding has ceased. The hand is supported by a pasteboard splint, and after a few days small compresses, wet with lead lotion placed between the fingers hasten the formation of skin over the wounded surfaces.

Subcutaneous division of the fascia palmaris is sometimes necessary to remove deformity produced by these adhesions.

It having been found that after simple division reunion was liable to take place by granulation from the palmar end of the incision, it was proposed by Rudttorfer to make an opening in the commissure, and when this had become cicatrized, to divide the remaining portion. The idea is clever, and the practice often successful. The commissure is perforated, and a piece of leaden wire is passed through and kept in the wound by being bent, until the edges of the opening have healed, then the remaining part of the commissure is divided, and the fingers bound with linen as before. Dieffenbach has not found this method so successful as others have done, the irritation produced by the lead leading to the necessity for its removal, and no formation of an open canal following.

In some cases, after division of the commissure, Dieffenbach has successfully united the edges of the wound made by this division by sutures, assisting the approximation of the edges by lateral incisions.

When the union is of the whole breadth of the finger, the only sure operation is the transplanting a flap of skin. The flap may be formed from the skin of the back of the hand, but it is apt to contract, the sutures suppurate, and the flap dies; and Dieffenbach, in all cases to which it is applicable, performs an operation which he describes as most successful, forming the flap from the commissure. We give the description in his own words:

"A long incision is made with a small pointed scalpel upon the back of the hand, at the border of the union, and of a finger which reaches to the middle of the posterior phalanx. A similar incision, parallel with the first, is made along the posterior part of the finger; then both incisions are united by a cross cut; then the anterior border of this strip of skin is fastened by hooked forceps, and loosened, with the greatest possible quantity of the subjacent cellular tissue, as far as the point where the fingers are normally separated. Its breadth, in an adult, should be a quarter of an inch; in children it is of course smaller. Then the points of the fingers are pressed apart from each other, and a long incision is made between the fingers through the skin, first on the back, and then a second longer one on the under surface reaching to the hand, and the remaining adhesion separated. On the volar aspect of the finger a cut, rather more than a quarter of an inch long, is made across before the end point of the incision; then the flap is turned in between the separated fingers, the epidermis surface thus remaining naturally turned outwards, and the anterior narrow end of the long four-cornered flap is fastened by three sutures to the wounded edge of the cross incision on the volar aspect. The remaining wounded edges of the fingers are brought towards each other by strips of plaster, and the space between the fingers is filled by soft charpie, and their points kept so far apart from each other by a thick compress, that the flap may not undergo the least pressure, and die from the approximation of the fingers; then the fingers are surrounded by a finger bandage, in order to retain the charpie, and the hand is fastened upon a splint.

"The neighbourhood of the flap, which must be free from the bandage, when inflammation comes on is covered by lead lotions, and the sutures are removed after its firm union; but still for a long time pressure is made upon the flap by small strips of plaster passing from the back of the hand between the fingers, reaching over the middle of the palm. The healing of the other wounds of the fingers follows without difficulty by granulation and cicatrization. If several fingers are united, the operation is repeated in the same manner at a subsequent period." (pp. 744-5.)

If the union be bony it may be divided after incision of the soft parts with a finger saw, but only in slight cases, taking care not to open the joints. In cases where bony union of all the fingers with each other exists operation is quite useless.

Operations upon adhering toes can be scarcely ever necessary, as the toes are as useful as when separated. If there is also contraction by which the anterior phalanx and nail are turned to the ground, subcutaneous division of the flexor tendon will be necessary.

In the following chapter a form of prominent ulcer is described frequently following loss of the toes, or their removal either at their articulation or in the middle of a phalanx. It is very obstinate, and causes great suffering. The means proposed for its cure are, after surrounding incisions and paring away the surface of the ulcer, cutting a proper sized flap from the back of the foot, covering the site of the ulcer with it by turning, and then uniting the edges by suture. We can say nothing on this point from personal observation.

MUSCLES AND TENDONS. A section of 100 pages on the division of tendons and muscles concludes this volume. In our Thirteenth Volume (pp. 1-28) we made a full analysis of the work of Dieffenbach on this express subject, and also of that of his follower and commentator, Phillips; and in our Eighth Volume, (pp. 385-405), of those of Stromeyer, Bouvier, and Little. Anything like a connected account, therefore, of the section before us is clearly unnecessary, and we shall conclude the present article by a few remarks upon such interesting points as strike us in perusal.

After the division of tendons, Dieffenbach much prefers flannel to linen bandages, being more elastic, and consequently impeding the circulation less should swelling come on.

With regard to the operations for stammering, he states that he operated in about eighty cases; a quantity (Anzahl) were completely cured; others which appeared to be cured commenced stammering again, some sooner, some later. Some upon whom the operation had apparently slight effect, became afterwards improved, but in by far the greater number, in whatever manner, or however often the operations were performed, they were ineffective. It is therefore only to be recommended in the most obstinate cases of stuttering, when exercises of the voice and other means have proved useless. It is a great pity that the exact number of those cured and benefited is not given. "A quantity," or "a certain number," is very indefinite.

The author's experience has convinced him that division of the muscles of the back, in cases of spinal curvature, is scarcely ever of any use.

When speaking of the division of muscles to favour the reduction of old dislocations he refers to a case which had resisted all attempts at replacement, and which readily yielded after subcutaneous division of the tendons of the pectoralis major and teres minor.

After division of the tendon achilles on account of contraction, the result of paralysis of the extensor muscles, extension must be used with great caution. Directly after division the foot can be brought to a right angle, and the end of the tendon separated some inches, and if this is done the space becomes filled by exudation of blood, and union does not take place. Fourteen days should be allowed to pass in such cases before the extension apparatus is applied, and the cure is complete a few weeks later.

We find nothing more which is not fully noticed in our former articles, with the exception of a few remarks on habitual spasm of the flexor pollicis longior. Many persons much accustomed to the use of the pen are subject to spasms in the muscles of the thumb and index finger, and the influence of the will over the direction of the pen is lost for the time, and they write quite a different hand to their ordinary character. Their writing appears as if they had been in a shaky carriage on a bad road. With time the spasms become more severe and constant, and shaking of the fingers, which may be compared to stuttering, comes on; at a more advanced stage the pen can only be held by passing it through a cork, and after a time even this becomes too small to be held. In one case the pen had to be passed through a cork from three to four inches square, and this was inclosed by the whole hand. Usual remedies are of no avail, except long continued disuse of the pen. In one case a patient who did not take a pen in his hand for six months, at the first trial afterwards wrote with great ease. With one sole exception the author has observed this condition in males only. He has practised tenotomy in such cases, but in only one was the cure perfect; in six others the state of things was the same after as before the operation. He divided the flexor pollicis brevis and longus, adductor pollicis, and the muscles of the index, according to the circumstances of the case, but without result. After the healing the spasms returned in the same degree, without any ill effect following, or the utility of the finger being much lessened.

NERVES. When we stated that the operative orthopedy concluded the volume, we had not observed a short chapter on the division of nerves. It is a very good one, giving a clear account of the experience of European surgeons, and taking a very unfavorable view of the operation under any circumstances. One case of the author, however, is interesting: very severe neuralgia had followed venesection; the injured nerve was divided, with instant effect, the pains, which had continued a month, immediately ceasing. A similar case is quoted from Hirsch, in which convulsions and coma accompanied local neuralgia, which were removed by two deep incisions over the wound. But whether for the relief of neuralgia, or tetanus, or for any other cause, success is rare; and "surgery is here under the greatest obligation to physiology, for pointing out that the sanguinary path is not the right one." The remedies the author has found most successful are the decoct. Gittmanni, iodine, and the cod-liver oil ("thrancur.")

Our analysis of this important work is now concluded. That its arrangement is faulty, and that for a complete system of operative surgery it is in many particulars defective, cannot be denied; still, in the departments of plastic surgery and tenotomy, it is by far the best work extant. The style is generally clear and forcible, and the descriptive portions, particularly when treating of the sufferings of patients or the results of operations, often border on the poetical. Still, in the description of many of the more delicate operations, the want of woodcuts in illustration is painfully felt. In more than one instance we had vainly endeavoured to satisfy ourselves that we had exactly comprehended the directions laid down, and in such cases have given a literal translation rather than a paraphrase, that we might not run the risk of misinterpretation. We would strongly advise the publication of a series of diagrams with the second volume, illustrating not only the operations to be there described, but also those included in the volume before us; the value of the work would then be greatly increased, and the student's task much facilitated.

Two courses were open to us in the composition of the preceding article: the one, to confine our attention to a very few subjects, and treat them fully by copious extracts and critical remarks; the other, to endeavour to give a general outline of the contents of the work before us, and a condensed account of its most important or new contents. We have adopted the latter, as likely to be the more useful; and as the work must become one of standard surgical reference, we shall hereafter have occasion to refer to it, when commenting on other works treating on any of the subjects we here have glanced over. To those who think we might have followed both courses, we would remark that it is not easy to condense eight or nine hundred pages of Dieffenbach's into forty or fifty of our own. This difficulty, however, is a test of the value of the work. We could point out effusions of similar extent in our own tongue, on which a single page would be fruitlessly expended. If writers would follow the example of our author, and keep silent until they had something valuable to communicate, much of the critic's task would be spared. It now only remains for us, in the name of our operating brethren, to return most cordial thanks to the veteran professor of Berlin for this most valuable contribution to the literature of our profession.

---

## Figures and Tables

**Figure f1:**